# Genetic Identification of a Network of Factors that Functionally Interact with the Nucleosome Remodeling ATPase *ISWI*

**DOI:** 10.1371/journal.pgen.1000089

**Published:** 2008-06-06

**Authors:** Giosalba Burgio, Gaspare La Rocca, Anna Sala, Walter Arancio, Dario Di Gesù, Marianna Collesano, Adam S. Sperling, Jennifer A. Armstrong, Simon J. van Heeringen, Colin Logie, John W. Tamkun, Davide F. V. Corona

**Affiliations:** 1 Dipartimento di Scienze Biochimiche, Universita' degli Studi di Palermo, Palermo, Italy; 2 Istituto Telethon Dulbecco, Universita' degli Studi di Palermo, Palermo, Italy; 3 Department of Molecular, Cell, and Developmental Biology, University of California Santa Cruz, Santa Cruz, California, United States of America; 4 Joint Science Department, Claremont McKenna, Scripps, and Pitzer Colleges, Claremont, California, United States of America; 5 Molecular Biology Department, Nijmegen Centre for Molecular Life Sciences, Radboud University Nijmegen, The Netherlands; European Molecular Biology Laboratory, Germany

## Abstract

Nucleosome remodeling and covalent modifications of histones play fundamental roles in chromatin structure and function. However, much remains to be learned about how the action of ATP-dependent chromatin remodeling factors and histone-modifying enzymes is coordinated to modulate chromatin organization and transcription. The evolutionarily conserved ATP-dependent chromatin-remodeling factor ISWI plays essential roles in chromosome organization, DNA replication, and transcription regulation. To gain insight into regulation and mechanism of action of ISWI, we conducted an unbiased genetic screen to identify factors with which it interacts *in vivo*. We found that ISWI interacts with a network of factors that escaped detection in previous biochemical analyses, including the Sin3A gene. The Sin3A protein and the histone deacetylase Rpd3 are part of a conserved histone deacetylase complex involved in transcriptional repression. ISWI and the Sin3A/Rpd3 complex co-localize at specific chromosome domains. Loss of ISWI activity causes a reduction in the binding of the Sin3A/Rpd3 complex to chromatin. Biochemical analysis showed that the ISWI physically interacts with the histone deacetylase activity of the Sin3A/Rpd3 complex. Consistent with these findings, the acetylation of histone H4 is altered when ISWI activity is perturbed *in vivo*. These findings suggest that ISWI associates with the Sin3A/Rpd3 complex to support its function *in vivo*.

## Author Summary

The eukaryotic genome is organized in a highly dynamic structure called chromatin. Access to DNA in the context of chromatin is granted by enzymatic activities that use the energy of hydrolysis of ATP to slide or covalently modify nucleosomes. ISWI is an evolutionarily conserved nucleosome-sliding factor that plays essential roles in transcription, DNA replication, and chromosome organization. Despite the wealth of data on ISWI function, little is known about how its activity is regulated and integrated in different physiological contexts *in vivo*. Using *D. melanogaster* as a model system, we conducted a genetic screen for factors regulating ISWI activity. One of the genes identified in our screen, *Sin3A*, encodes a subunit of a histone deacetylase complex that may regulate ISWI function by modifying its nucleosome substrate. Our genetic screen revealed that *ISWI* interacts with a network of cellular and nuclear factors that escaped previous biochemical analyses, indicating the participation of ISWI in a variety of biological processes not linked to date with known ISWI functions.

## Introduction

Eukaryotic cells store their genetic information in the form of chromatin, a complex of DNA packed with structural and regulatory proteins. The functional repeating unit of chromatin is the nucleosome, 146 base pairs of DNA wrapped around an octamer of histone proteins. While this packaging provides the cell with the obvious benefit of organizing a large and complex genome in the nucleus, it can also block access to DNA. Nuclear reactions therefore depend on factors that modulate the accessibility of DNA within the context of chromatin. ATP-dependent chromatin remodeling and the covalent modification of histone amino termini, play central roles in determining chromatin accessibility [1]–[3]. These reactions are catalyzed by evolutionarily conserved multi-subunit chromatin-remodeling complexes that directly alter chromatin structure to regulate gene expression and other nuclear functions [1]–[4].

ISWI is a component of several ATP-dependent chromatin remodeling complexes conserved in composition and function across species [5],[6]. In higher eukaryotes, ISWI is an abundant and ubiquitously expressed protein that is essential for cell viability [7],[8]. In humans, the loss of ISWI function is associated with the multi-systemic disease Williams-Beuren syndrome [9]–[11]. In *Drosophila*, ISWI is the ATPase subunit of three chromatin remodeling complexes: NURF (NUcleosome Remodeling Factor), ACF (ATP-utilizing Chromatin assembly and remodeling Factor) and CHRAC (CHRomatin Accessibility Complex) [5],[6]. *In vitro*, ISWI uses the energy of ATP hydrolysis to catalyze nucleosome spacing and sliding reactions [12]. Loss of ISWI function in *Drosophila* results in dramatic chromosome condensation defects and in reduction of chromatin-bound histone H1 levels, suggesting that ISWI plays a general role in chromosome condensation *in vivo* by promoting the loading of the linker histone H1 on chromatin [7],[13].

Genetic and biochemical studies have also supported a role for ISWI in promoting transcription [7],[14],[15]. However, the preferential association of ISWI with transcriptionally silent chromatin, together with the changes in gene expression in *Drosophila ISWI* mutants suggest that ISWI plays an important role in transcriptional repression [7],[13]. The yeast ISWI homologs Isw1 and Isw2 are subunits of multi-subunit complexes involved in transcription activation and repression [16]. The mammalian ISWI homolog, SNF2H, is part of the nucleolar remodeler NoRC complex, that has been shown to be involved in the repression of Pol I-dependent transcription [17],[18]. Therefore, ISWI family complexes appear to both activate and repress transcription. Studies in several model organisms have implicated ISWI in a variety of other nuclear functions including DNA replication, telomere silencing, stem cell self-renewal and nuclear reprogramming [5],[19].

Nucleosome spacing reactions catalyzed by ISWI can be regulated by its associated subunits. ACF1, a subunit of the ACF complex, modulates ISWI enzymatic functions both quantitatively and qualitatively and targets ISWI to heterochromatic replication sites *in vivo* [20]–[23]. Similarly, the NURF301 protein facilitates nucleosome remodeling by ISWI [24],[25] and the CHRAC-specific subunits CHRAC14 and CHRAC16 improve the efficiency of ISWI-mediated nucleosome sliding [26],[27]. Interestingly, the non-histone protein HMGB1 accelerates the nucleosome sliding activity of ISWI, probably acting as a chaperone in the rate-limiting DNA distortion step occurring during nucleosome sliding [28].

The ability of ISWI complexes to remodel chromatin can also be influenced by the covalent modification of their nucleosomal substrate. For example, the nucleosome remodeling activity of ISWI is counteracted *in vivo* and *in vitro* by the acetylation of histone H4 on Lys16 [29]–[31]. Consistent with the critical role of the histone H4 tail in nucleosome recognition, the acetylation of histone H4 on Lys12 and Lys16 impairs substrate recognition by ISWI [32]. By contrast, tri-methylation of histone H3 on lysine 4, a mark of active transcribed genes, recruits the human NURF complex to Hox gene promoters to maintain their expression patterns during development [24]. Moreover, the di- and tri- methylation of histone H3 on lysine 4 can recruit the yeast Isw1 protein to certain genes, to regulate the association of Pol II with the coding regions [33]. Recently, it has been also reported that the histone acetyltransferase GCN5 can acetylate ISWI itself at the conserved lysine K753, though the biological significance of this modification needs further investigation [34].

Due to the broad spectrum of functions played by ISWI, it is likely that other factors or histone modifications may influence its activity *in vivo*. In order to identify new regulators of ISWI function, we conducted an unbiased genetic screen for dominant enhancers of phenotypes caused by loss of ISWI function in *D. melanogaster*. One class of mutants isolated in the screen includes factors that covalently modifying histones. Among this class we found mutants in the gene encoding Sin3A, a component of a conserved transcriptional repression complex containing the histone deacetylase protein Rpd3 [35]. Immunostaining showed that ISWI and the Sin3A/Rpd3 complex partially co-localize on polytene chromosomes and that Sin3A and Rpd3 levels are reduced on *ISWI* mutants chromosomes. Immunoprecipitation experiments showed that ISWI physically interacts with both Sin3A and Rpd3. Chromatographic purification of larval nuclear extracts revealed that ISWI is associated with a histone H3/H4 deacetylase activity. Furthermore, the distribution and levels of histone H4 acetylation change when ISWI activity is altered. Our data support a model in which ISWI recruits the Sin3A/Rpd3 complex to specific chromosome domains. Given the functional antagonism between ISWI and site-specific histone acetylation [29]–[32], we propose that the Sin3/Rpd3 complex cooperates with ISWI to deacetylate histone and facilitate ISWI chromatin remodeling activity *in vivo*.

## Results

### A Genetic Assay for Identifying Factors that Interact with *ISWI In Vivo*

Several features of the *Drosophila* eye make this tissue particularly appealing for genetic studies of complex biological processes [36]. The misexpression of dominant-negative alleles of chromatin-remodeling enzymes in the eye-antennal disc can compromise eye development, often causing roughness and/or reduced eye size [37]. This approach has been successfully used to conduct a genetic screen for modifiers of phenotypes caused by loss of the chromatin-remodeling factor *brm* [37],[38]. *ISWI* mutations are lethal in *Drosophila* [7], highlighting the utility of this approach in the identification of components that regulate its function *in vivo.*

**Figure 1:**
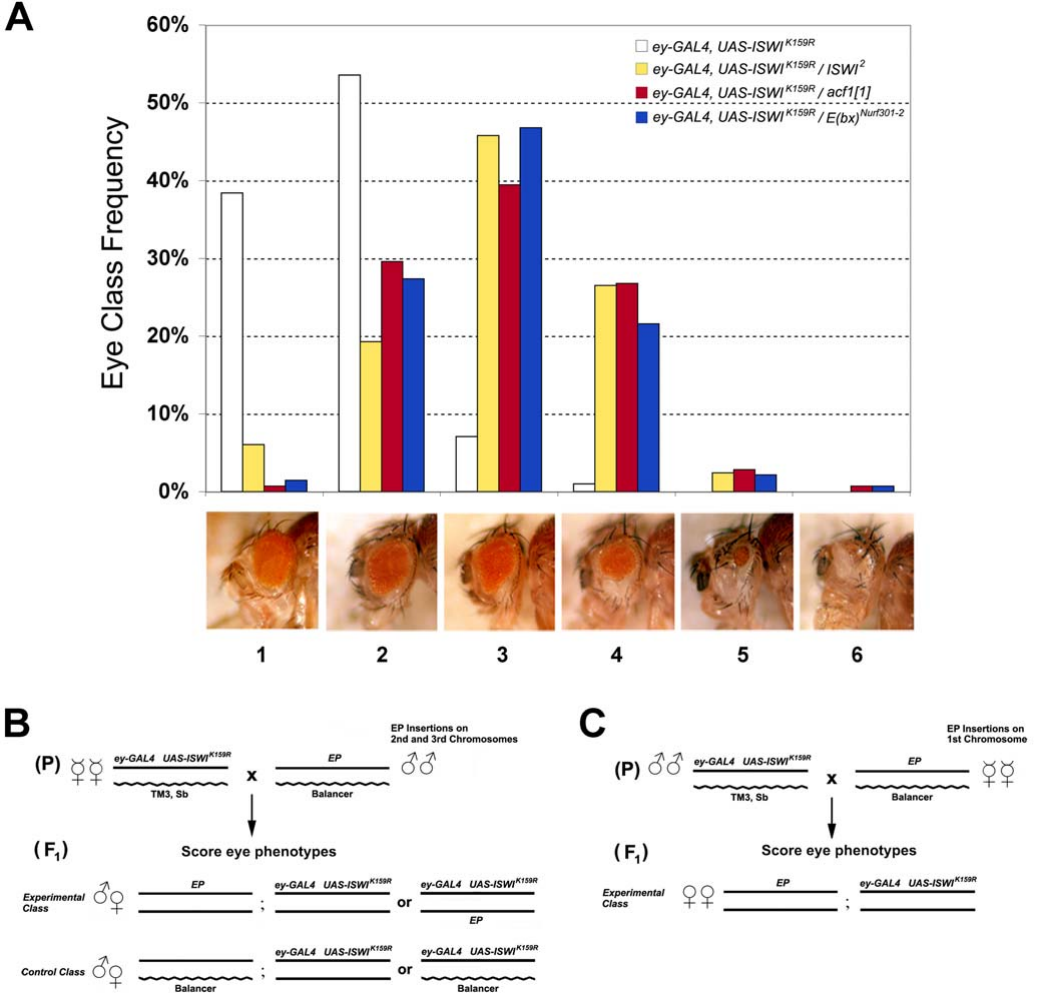
An eye assay to identify modifiers of *ISWI^K159R^.* (A) We assigned individual eyes a score from 1 to 6 based on the severity of the defects [37],[38]. Similarly to the *ISWI^2^* allele [7], null mutations for *acf1* and *E(bx)* strongly enhanced eye defects caused by expression of the *ISWI^K159R^* transgene. Our scoring system allowed the visualization of *ISWI^K159R^* eye defects as phenotypic class distributions, helping the identification of modifiers of *ISWI^K159R^* phenotypes that are statistically significant. (B) Outline of the F_1_ eye-based screen for dominant modifiers of *ISWI^K159R^* by EP insertions on the second, third and (C) X chromosomes.

A single K159R amino acid substitution in *Drosophila* ISWI (ISWI^K159R^) eliminates its ATPase activity, without affecting the ability of the mutant protein to be incorporated into native complexes [7],[39]. As previously reported, the expression of a *UAS-ISWI^K159R^* transgene in the developing eye, using an *ey*-GAL4 driver, has strong effects on cell viability and chromosome organization and results in flies with rough and reduced eyes (Figure S1) [7],[13],[29]. We reasoned that mutations that enhance or suppress phenotypes resulting from the expression of ISWI^K159R^ are likely to define genes involved in the same biological process as *ISWI* [37],[38]. Indeed, elevated expression of *Drosophila* MOF, which counteracts ISWI activity by acetylating histone H4 on lysine 16, enhances *ISWI^K159R^* eye phenotypes [29]. To further validate this approach we checked whether mutations in genes that encode proteins that are known to physically interact with ISWI, like *acf1* and *E(bx)*, enhance *ISWI^K159R^* eye phenotypes. Indeed, null mutations in *acf1* and *E(bx)* strongly enhanced eye defects caused by *ISWI^K159R^* (Figure 1A). These genetic interactions are specific since the *acf1* and *E(bx)* null alleles did not enhance eye defects caused by the misexpression of a dominant-negative form of another chromatin remodeler, *brm^K804R^ (*data not shown).

### Isolation of *ISWI^K159R^* Modifiers

To facilitate systematic dominant-modifier screens in *Drosophila*, a collection of ∼2300 *Drosophila* lines bearing EP elements has been generated [40],[41]. EP elements are modified transposable P-elements that may lead to the interruption of a gene or to GAL4-dependent misexpression of the genes adjacent to the insertion site. The EP elements of this collection have been precisely mapped and genes corresponding to the interacting modifier lines can be easily identified by their annotation on *Flybase* (www.flybase.org). Therefore, we screened the entire EP collection because of the potential to identify both gain and loss of function mutations in known genes.

The F_1_ progeny of crosses between males bearing EP elements and females of the sensitized line expressing *ISWI^K159R^* under the control of the *eyGAL4* driver were screened for enhancers or suppressors of *ISWI^K159R^* eye defects (Figure 1B & C). Although we employed conditions to isolate both enhancers and suppressors, our genetic screen resulted in the exclusive isolation of enhancers of *ISWI^K159R^* eye phenotypes. One class of false positives that we expected to recover corresponds to EP elements that cause GAL4-dependent eye defects independently of the *ISWI^K159R^* transgene. This class of false positives was identified by directly crossing the interacting EP lines with flies carrying only the *eyGAL4* driver (Table S1B) and corresponded to the EP lines identified independently in a previous genetic screen for *eyGAL4* specific eye defects [42].

Another class of false positives that we expected were mutations that altered the expression of the *UAS-ISWI^K159R^* transgene or modify phenotypes resulting from its expression by altering cell viability or proliferation. To exclude such mutations, we assayed interactions between the EP elements and, *brm^K804R^*, a dominant-negative form of the chromatin remodeling factor BRM. The *eyGAL4*-dependent misexpression of *brm^K804R^* causes eye defects similar to the *ISWI^K159R^* transgene [37],[38]. We screened the entire EP collection for insertions that modified eye defects caused by misexpression of *brm^K804R^* (Table S1A) and considered insertions as *bona fide ISWI^K159R^* modifiers only if the EP line specifically enhanced *ISWI^K159R^* but not *brm^K804R^* eye phenotypes (Table S1A). The *ISWI^K159R^* enhancer EP line insertions that passed the above secondary screens were classified according to their strength of interaction (Table S1A), as described in methods.

### Gene Ontology and Interaction Network Analysis of *ISWI^K159R^* Enhancers

Our eye scoring system allowed the visualization of *ISWI^K159R^* eye defects as phenotypic class distributions. Using data available on Flybase (www.flybase.org) we mapped *ISWI^K159R^* enhancers to 255 protein coding loci (Figure S2A & Table S1A) and found 21 strong (∼1% of total EPs screened), 65 medium (∼4%) and 171 weak (∼7%) *ISWI^K159R^* enhancers (Figure S2B). Our scoring strategy defined different stringency thresholds for the *ISWI^K159R^* enhancers. One advantage of this approach is that the *ISWI^K159R^* enhancers can be processed and analyzed independently as three distinct classes with three levels of interaction stringency. Furthermore, this semi-quantitative classification allowed us to isolate many weak interactions that often map in genes for which we also recovered strong or medium *ISWI^K159R^* enhancers, helping us to focus on mutants that are bona fide *ISWI^K159R^* interactors, as defined by the recovery of multiple independent insertions in the same gene (Table S1C).

In order to understand the biological processes regulated by the *ISWI^K159R^* enhancers we isolated, we conducted a Gene Ontology analysis [43],[44] employing the latest protein annotations available at Flybase (Release FB2007_03; www.flybase.org). When compared to the entire fly proteome, the EP line collection proved to be significantly enriched for a number of GO categories (Figure S3A). Therefore, we analyzed the genes corresponding to *ISWI^K159R^* enhancers using the entire EP library as reference population rather than the entire *Drosophila* proteome. Although most subcellullar localizations were represented normally relative to the starting EP collection, we found that *ISWI^K159R^* enhancers were over represented in nuclear (n = 65, P value = 0.04) but not in cytoplasmic factors (n = 18, P value = 0.04) (Figure S2C). Interestingly, in depth analysis revealed that the strong *ISWI^K159R^* enhancers showed a significant enrichment for regulators of the cell cycle, and in particular for negative regulation of the cell cycle (*pum, grp, trbl, abl, pbl, pnut*, P value = 0.06) (Figure S3B, upper panel). Furthermore, when combined, the medium and strong *ISWI^K159R^* enhancers showed a significant enrichment for the biological processes ‘neuron differentiation’ and ‘neuron development’ (*ttk, acf1, Sin3A*; P value = 0.03) (Figure S3B, lower panel). This enrichment is consistent with the recent recovery of *ISWI* and several *ISWI^K159R^* enhancers (e.g.: *E(bx*), *mbf1)* in a genetic screen for factors required for sensory neuron morphogenesis [45].

In order to gain an integrated view of the functional network of the genes isolated in our screen we mapped all known genetic and physical interactions existing within the *ISWI^K159R^* enhancers with the help of the BioGrid. The BioGrid is an integrated database containing the information on all known physical and genetic interactions for a variety of model organisms, including *D. melanogaster* [46] (http://www.thebiogrid.org/). Our analysis revealed that some of the *ISWI^K159R^* enhancers interact with each other, emphasizing the functional correlation existing between some of the factors isolated in our screen (Figure S2C). One major hub of genetic interactions we found amongst the *ISWI^K159R^* enhancers is represented by the *Ras85D* gene, which is known to interacts with 131 fly factors [46], of which 48 are represented by EP lines, 12 of which were recovered in our *ISWI^K159R^* screen (Figure S2C). This result is consistent with a recent report showing genetic interactions between the *C.elegans ISWI* gene, *ISW-1*, and the RAS pathway in the regulation of vulval cell fates [47].

### ISWI Genetically Interacts with *Sin3A* and *Rpd3*

**Figure 2:**
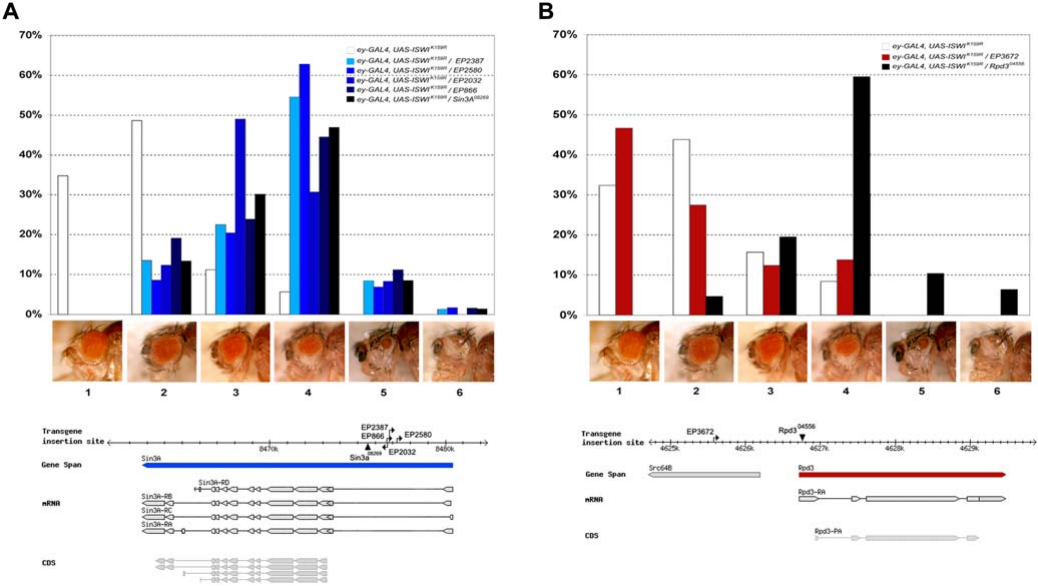
Genetic interaction of *ISWI* with *Sin3A* and *Rpd3.* Loss of Sin3A or Rpd3 function enhances defects caused by the expression of *ISWI^K159R^* in the eye. (A) Four EP insertions mapping on the first intron shared by the multiple transcripts of the Sin3A gene were recovered in our screen. Interactions of *EP2387*, *EP2580*, *EP866*, *EP2032* and the null *Sin3A^08269^* allele with *ISWI^K159R^* are shown. (B) The null Rpd3^04556^ allele, but not *EP(3)3672* inserted ∼10Kbp away from the *Rpd3* in the *Src64B* gene enhances *ISWI^K159R^*. The top panels show a graphical histogram representation of the eye scoring data reported in Table 1. The lower panels show genomic mapping data for the EP’s (directional arrows) and *Sin3A*, *Rpd3* alleles (arrowheads) tested.

We decided to focus our initial analysis on *ISWI^K159R^* genetic interactors that could play a direct role in the regulation of ISWI *in vivo.* We therefore ignored genes that are regulated by ISWI [13] or that genetically interacted with the chromatin-remodeling factor *brm* (Table S1A). Among these genes, we further restricted our attention to enhancers encoding chromatin components that were identified by multiple EP hits and that were part of highly correlated interaction networks. One gene that fit these criteria is *Sin3A*. The *Sin3A* gene is not a transcriptional target of ISWI (Table S1A) [13], it is part of a highly connected network of *ISWI^K159R^* enhancers (Figure S2C), and four independent EP insertions in the *Sin3A* gene enhance *ISWI^K159R^* eye phenotypes (Figure 2A & Table S1C). The Sin3A protein is generally associated with highly conserved protein complexes containing the histone deacetylase Rpd3 protein [35]. Previous work in *S.cerevisiae* has shown that *Sin3A* and *Rpd3* genetically interact with the *ISWI* homolog *Isw2* [48],[49]. One of the goals of our genetic screen was to identify nuclear enzymatic activities that could regulate ISWI function in higher eukaryote chromatin. Therefore, we decided to investigate whether the evolutionary conserved genetic interaction existing between *ISWI* and *Sin3A* was connected to ISWI regulation by histone acetylation in the higher eukaryote *D. melanogaster*.

All four *Sin3A* EP enhancer insertions map to the first intron of the *Sin3A* gene (Figure 2A). While the EP2032 insertion, whose orientation may potentially drive the misexpression of *Sin3A*, weakly interacts with *ISWI^K159R^*; the EP2580, EP2387 and EP866 lines are inserted as to direct anti-sense transcription and strongly enhance *ISWI^K159R^* eye phenotypes (Figure 2A & Table 1). Furthermore, a null allele of the *Sin3A* gene, *Sin3A^08259^,* also strongly enhanced *ISWI^K159R^* (Figure 2A & Table 1), indicating that loss of *Sin3A* function enhances eye defects resulting from the expression of *ISWI^K159R^*.

**Table 1:** Scoring of *ISWI^K159R^* eye phenotype enhancements caused by EP insertions on the *Sin3A* and *Rpd3* genes.

Progeny expressing	Eye Score						*P-value*	Total Number of Eyes
	1	2	3	4	5	6		
*Df(yw)*	25	35	8	4	0	0	NA	72
*Sin3A^08269^, cn1*	0	19	43	67	12	2	<0.001	143
*CyO, ry^[506]^*	37	55	18	13	2	0		125
*EP2387*	0	21	35	85	13	2	NA	156
*EP2580*	0	5	12	37	4	1	<0.001	59
*CyO*	19	27	12	5	0	0		63
*EP2032*	0	6	24	15	4	0	<0.001	49
*CyO*	12	23	12	2	0	0		49
*EP866*	0	12	15	28	7	1	<0.001	63
*CyO*	17	25	10	3	0	0		55
*Rpd3^04556^, ry^[506]^*	0	8	34	104	18	11	<0.001	175
*TM3, ry^RK^, Sb^1^, Ser^1^*	31	42	15	8	0	0		96
*EP3672*	34	20	9	10	0	0	NA	73

**Figure 3:**
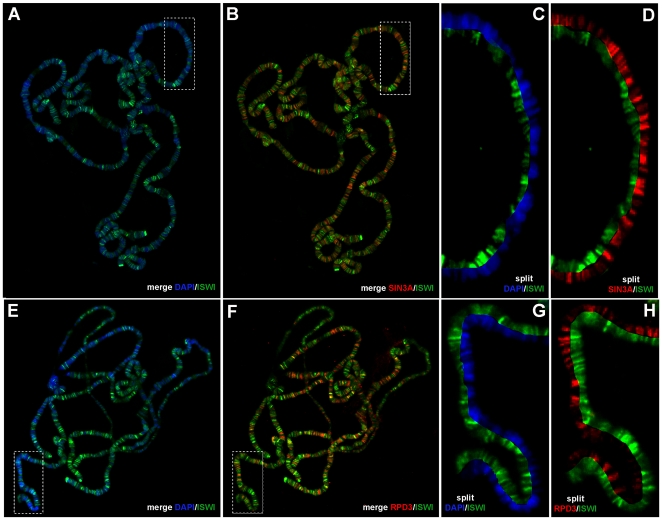
ISWI co-localize with Sin3A and Rpd3 at many sites on polytene chromosomes. (A, C, E and G) Distribution of ISWI and DAPI or (B and D) ISWI and Sin3A or (F and H) ISWI and Rpd3 proteins on salivary gland polytene chromosomes from wild-type third instar larvae. (C, D, G and H) Magnifications of boxed areas shown as “split” chromosome images. Displaying multiple staining patterns in a ‘‘split’’ format helps avoiding visual artifacts when high levels of one protein can mask low levels of another [37],[38]. ISWI, while present at varying abundances it co-localize with Sin3A and Rpd3 proteins at many sites on polytene chromosomes.

Table of data used to generate graphs shown in Figure 3. When control progeny class data were available, the Kolmogorov-Smirnov two-sample test was applied to calculate if the cumulative frequency distributions of the eye scores of the experimental and control progeny classes were statistically different (P = 0.05).

The Rpd3 histone deacetylase physically interacts with the Sin3A protein in flies [35]. We therefore used our eye assay to test the interaction of a null allele of *Rpd3* (*Rpd3^04556^*), and re-tested the only insertion close to the *Rpd3* gene available from the EP collection, the EP3672 line (Figure 2B). The *Rpd3^04556^* allele strongly enhanced *ISWI^K159R^* eye phenotypes while the EP3672 insertion did not (Figure 2B & Table 1), probably because the EP3672 allele would drive rather than reduce the expression of the *Rpd3* gene. Our data indicate that a reduction in the level of either of the two central subunits of Sin3A/Rpd3 histone deacetylase complex enhances *ISWI^K159R^* phenotypes in the developing eye.

### The Sin3A/Rpd3 Complex and ISWI Partially Co-Localize on Polytene Chromosomes

If Sin3A and Rpd3 work in concert with ISWI we would expect them to bind common chromatin target sites. Thus, we looked at the relative distribution of ISWI and the Sin3A/Rpd3 proteins on *Drosophila* salivary gland polytene chromosomes by indirect immunofluoresence microscopy using antibodies against ISWI and Sin3A. ISWI preferentially binds to DAPI stained bands, though a significant fraction of the protein is also associated with interbands or the interface between bands and interbands (Figure 3C & G). At first glance, the ISWI and Sin3A proteins appeared to bind polytene chromosomes in a non-overlapping pattern (Figure 3B). However, when we “split” images of chromosomes down the middle to directly compare ISWI and Sin3A binding [37],[38] we observed that ∼50% of ISWI bands co-localize with Sin3A (Figure 3D). The Sin3A and Rpd3 binding patterns on salivary gland polytene chromosomes are highly coincident [35] and, as expected, ∼40% of ISWI bands also co-localize with Rpd3 (Figure 3F & H). Thus, while ISWI and the Sin3A/Rpd3 complex tend to bind chromosome sites with different relative abundances, they do overlap at many sites on polytene chromosomes.

### ISWI Physically Interacts with the Sin3A/Rpd3 Complex

**Figure 4:**
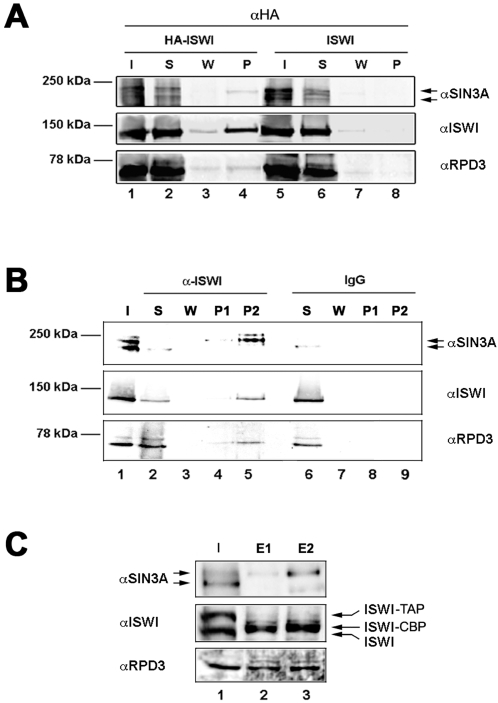
ISWI physically interacts with Sin3A and Rpd3 in embryo and larval stages. (A) Immunoprecipitation with anti-HA antibodies using embryo protein extracts derived from a line expressing HA-tagged ISWI (HA-ISWI) and from control extracts (ISWI). ISWI is specifically immunoprecipitated from the HA-ISWI extract together with the Rpd3 and Sin3A proteins. (B) Immunoprecipitation with the anti-ISWI antibody, but not with control IgG, immunoprecipitates Sin3A and Rpd3 on embryo protein extracts. Western blot analysis was performed on 10% of the total input extract [I], supernatant [S], wash [W], 5% of the total pellet [P1], and 30% of the total pellet [P] and [P2] using antibodies against ISWI, Sin3A and Rpd3. The arrows indicate two Sin3A isoforms detected by Western. (C) Larval nuclear extracts derived from larvae expressing TAP-tagged ISWI were bound to IgG Agarose beads. The ISWI-CBP fusion, consisting of the ISWI protein fused in frame with the calmodulin binding peptide, was eluted from the resin by cleavage with the TEV protease. The ISWI-CBP fusion protein eluted together with Sin3A and Rpd3 in nuclear extracts purified from larvae expressing TAP-tagged ISWI. The TEV cleaved eluate was rebound to a calmodulin coupled resin and the bound proteins were eluted by stripping the resin with SDS-loading buffer. Densitometric analysis indicate that ∼15% of total Sin3A and Rpd3 present in larval nuclear extracts associate with ISWI. Western blot analysis was performed on 0.05% of the total input extract [I], 3% of the TEV cleaved eluate [E1] and 3% of the SDS stripped eluate [E2], using antibodies against ISWI, Sin3A and Rpd3. Arrows indicate TAP-tagged ISWI [ISWI-TAP], ISWI fused in frame with the calmodulin binding peptide [ISWI-CBP] and endogenous untagged ISWI [ISWI].

The binding of ISWI, Sin3A and Rpd3 to many common sites suggested that the three proteins might physically interact. To test this possibility, we examined whether Sin3A and Rpd3 co-immunoprecipitate with ISWI from embryo extracts derived from flies expressing HA-tagged ISWI under the control of the natural ISWI promoter [7]. The anti-HA antibody specifically immunoprecipitated ISWI from extracts containing HA-tagged ISWI, but not from control extracts lacking the *HA-ISWI* transgene (Figure 4A, lanes 4 & 8). Consistent with a physical interaction with ISWI, a small but reproducible amount of both Sin3A and Rpd3 was detected in the pellet immunoprecipited from the HA-ISWI protein extracts, but not from control extracts (Figure 4A, lanes 4 and 8). Affinity-purified antibodies against ISWI specifically co-immunoprecipitated both the Sin3A and the Rpd3 proteins (Figure 4B, lanes 4,5,8 and 9), providing further evidence that the Sin3A/Rpd3 complex is physically associated with ISWI in *Drosophila* embryos. The antibody directed against the Sin3A protein recognizes two isoforms in embryo extracts, running at about ∼200 kDa and 220 kDa, as previously reported [35]. Interestingly, our data suggest that ISWI is physically associated with the higher molecular weight form of Sin3A (Figure 4A, lanes 1 and 4: Figure 4B, lanes 1, 4 and 5).

Since our genetic assay detected interactions between ISWI and other factors in the eye-antennal imaginal disc, we extended our biochemical studies to larval tissues. As predicted by the partial co-localization of ISWI, Sin3A and Rpd3 on polytene chromosomes (Figure 3), we also found that the three proteins can be co-immunoprecipitated from extracts of larval salivary glands (Figure S5A). Making use of a protocol, we recently developed to produce highly stable native nuclear extracts from whole larvae [50], we also conducted Tandem Affinity Purification (TAP) [51] of ISWI and associated proteins from larval nuclear extracts. TAP-tagged ISWI was affinity purified from a fly line where the *UAS-ISWI-TAP* transgene was expressed under the control of the *Act5C*-GAL4 driver. The Act5C-GAL4 dependent expression of the *UAS-ISWI-TAP* transgene can rescue the lethality associated with the *ISWI^1^/ISWI^2^* trans-heterozygous mutant, indicating that the presence of the TAP-tag does not interfere with the ISWI-TAP protein fusion function *in vivo* (data not shown). Following the two affinity purification steps, using larval nuclear extracts expressing the ISWI-TAP protein, ISWI tagged with the calmodulin binding peptide (ISWI-CBP) was successfully eluted by TEV cleavage (Figure 4C, lane 2) and remained stably associated with the calmodulin resins in the second affinity step together with Sin3A and Rpd3 proteins (Figure 4C, lane 3). These data indicate that a fraction of ISWI and the Sin3A/Rpd3 complex are physically associated during larval development.

Given the genetic and physical interaction we found between *ISWI*, *Sin3A* and *Rpd3* and their co-localization at many sites on polytene chromosomes, we asked whether the nucleosome stimulated ATPase ISWI and the histone deacetylase (HDAC) complex Sin3A/Rpd3 might be part of a yet unidentified multi-subunit complex. Interestingly, a fraction of ISWI from wild-type larval nuclear extracts strongly binds to nickel-coupled affinity resins (data not shown). The size fractionation of highly enriched larval nuclear proteins eluted from a nickel resin indicate that ISWI is part of a high molecular weight complex of about 600 KDa (Figure S4A). The Sin3A and Rpd3 proteins also elute with a profile similar to ISWI (Figure S4A). Although, ISWI and the Sin3A/Rpd3 complex can independently form large complexes that co-purify, the co-elution observed by gel filtration is at least in part due to direct physical interaction because ISWI and Sin3A/Rpd3 co-immunoprecipitate in these fractions (Figure S5E).

### The ISWI ATPase Is Associated with an HDAC Activity

**Figure 5:**
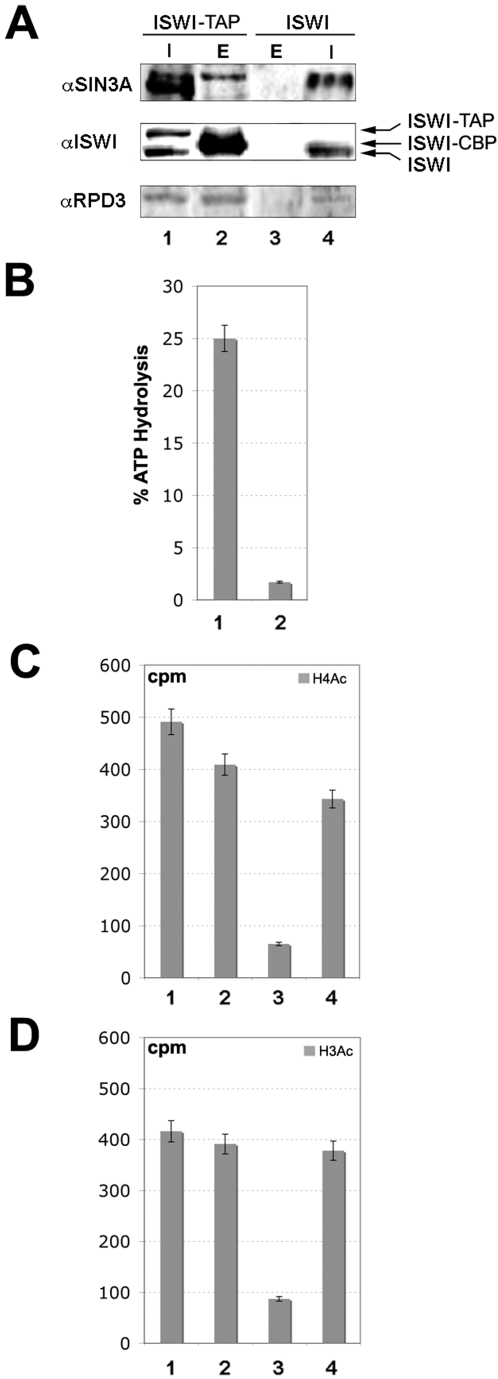
ISWI is associated with an HDAC activity co-eluting with Sin3A and Rpd3. (A) ISWI together with Sin3A and Rpd3 specifically eluted by TEV cleavage from larval nuclear extracts derived from the TAP-tagged ISWI line [ISWI-TAP], but not in control untagged extracts [ISWI]. Proteins eluting by TEV cleavage, containing ISWI-CBP together with Sin3A and Rpd3 were assayed for (B) nucleosome-stimulated ATPase and (C & D) HDAC activity on acetylated histone H4 and H3 substrates. The fraction containing ISWI-CBP [lane 1] but not the control fraction [lane 2] has nucleosome stimulated ATPase. A specific histone H4 and H3 HDAC activity was also found associated with the fraction containing ISWI-CBP [lane 2] but not the control fraction [lane 3]. For the ATPase assay, about 1.5% of Input [I] and TEV cleaved eluates were tested for ATPase activity in the presence of 100 ng of reconstituted recombinant chromatin. The HDAC assays were conducted on 15000 cpm of acetylated histones, with 8% of Input [I] and TEV cleaved eluates.

We next assayed the affinity purified TAP-tagged ISWI eluate and the gel filtration fractions containing ISWI, Sin3A and Rpd3 for nucleosome-stimulated ATPase and HDAC activities. As expected TAP-tagged ISWI (Figure 5A & B) and the gel filtration fractions enriched for ISWI (Figure S4A & B) showed nucleosome-stimulated ATPase activity. Consistent with a physical interaction between ISWI and the Sin3A/Rpd3 complex, an HDAC activity specific for *in vitro* acetylated histone H3 and H4 substrates (Figure S5B, C & D) co-eluted with the affinity purified TAP-tagged ISWI eluate (Figure 5C & D) and in the gel filtration fractions enriched for ISWI, Sin3A and Rpd3 (Figure S4 C & D). Thus, our data strongly suggest that the physical association between ISWI, Sin3A and Rpd3 couples the nucleosome-stimulated ATPase activity of ISWI with the HDAC activity of the Sin3A/Rpd3 complex.

### Loss of *ISWI* Causes a Reduction in the Levels of the Sin3A/Rpd3 Complex Bound to Chromatin

The recruitment of the Sin3A/Rpd3 histone deacetylase complex to chromatin through the physical interaction with ISWI, may explain their co-localization at specific loci on salivary gland polytene chromosomes. Alternatively, ISWI and the Sin3A/Rpd3 complex could bind the same chromatin domains independently. If ISWI plays a role in loading the Sin3A/Rpd3 complex on chromatin, we expect that loss of chromatin-bound ISWI should determine a change in the binding or distribution of Sin3A and Rpd3 on polytene chromosomes. To test if ISWI plays a role in the recruitment of the Sin3A/Rpd3 complex, we compared the relative levels of the Sin3A and Rpd3 proteins on wild type and *ISWI* mutant chromosomes.

**Figure 6:**
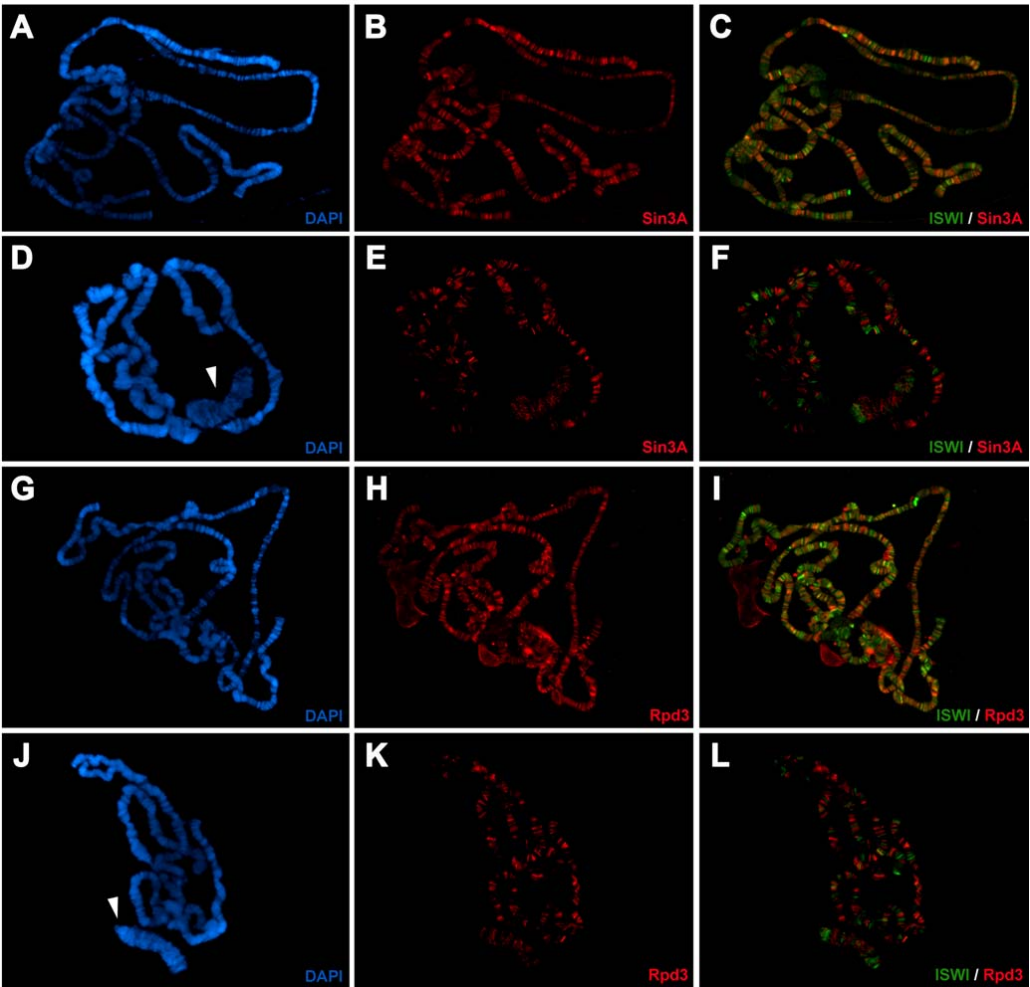
Loss of ISWI reduces the binding of Sin3A and Rpd3 to chromatin. (A and G) DAPI and immunostaining of (B) Sin3A or (H) Rpd3 on wild-type salivary gland male polytene chromosomes. (D and J) DAPI staining and distribution of (E) Sin3A or (K) Rpd3 on *ISWI* mutant salivary gland male polytene chromosomes. Merge of a double immunostainig for ISWI and Sin3A (C and F) or ISWI and Rpd3 (I and L) on wild-type and ISWI mutant salivary gland male polytene chromosomes, respectively. *ISWI* mutant chromosomes show a significant reduction in the levels of chromatin bound Sin3A and Rpd3 proteins when compared to wild-type chromosomes. The arrows mark the male X chromosome.

Salivary gland polytene chromosomes of *ISWI* mutant larvae show a partial reduction in the level of binding of both Sin3A and Rpd3 compared to wild-type chromosomes (Figure 6B, E, H and K). This is not due to the decreased expression of either protein in ISWI mutants, since the Sin3A and Rpd3 genes are not under the transcriptional control of ISWI [13] and their protein levels are not reduced in *ISWI* mutant salivary glands (Figure S6A). Indeed, Sin3A protein levels are actually higher in *ISWI* mutants as compared to wild type salivary glands (Figure S6A). Furthermore, the binding of Mod, a chromatin protein whose binding is particularly sensitive to chromosome morphology [52],[53], does not significantly change in wild-type and *ISWI* mutant polytene chromosomes, indicating that the effects we observed are probably not due to general chromosome condensation defects (Figure S6B).

In order to directly measure the Sin3A and Rpd3 binding on polytene chromosomes we quantified the relative signal resulting from double immunostainings for Sin3A/Mod and Rpd3/Mod in wild type and *ISWI* mutant chromosome, using the Mod staining as internal control [52],[53]. Our analysis revealed that on average there is a ∼36% and ∼48% reduction for Rpd3 and Sin3A chromosome binding in *ISWI* mutant chromosomes, respectively. Despite the significant reduction in Sin3A and Rpd3 binding, many residual sites of Sin3A and Rpd3 binding are observed in ISWI mutants (Figure 6E & K). We therefore compared the distribution of ISWI to Sin3A and Rpd3 on the polytene chromosomes of ISWI mutants using double-label immunofluoresence microscopy. Interestingly, the residual Sin3A and Rpd3 bands on *ISWI* mutant chromosomes do not co-localize with the residual ISWI of maternal origin [13], suggesting that they represent sites where Sin3A and Rpd3 are recruited to chromatin via ISWI-independent mechanisms. Taken together, our data suggest that ISWI could target the HDAC activity associated with the Sin3A/Rpd3 complex to specific chromatin domains, though Sin3A and Rpd3 are recruited via ISWI-independent mechanisms at many chromatin sites.

### Changes in the Levels of ISWI Cause Alterations in Histone Acetylation Chromatin Patterns

**Figure 7:**
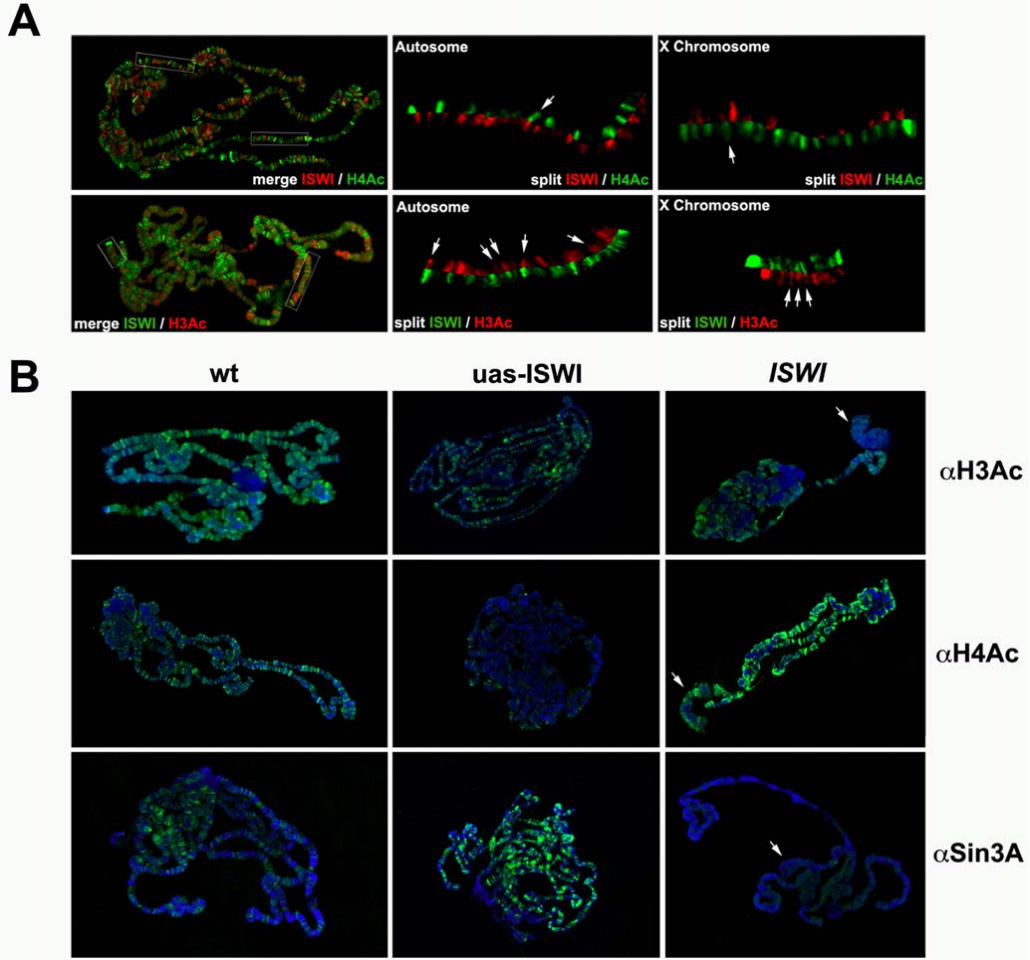
Changes in chromatin bound ISWI affects the levels of acetylated histone H3 and H4. (A) Immunofluorescence detection of ISWI [red] and acetylated histone H4 [green; K5Ac, K8Ac, K12Ac, K16Ac] on wild-type salivary gland polytene chromosomes [upper panel]. The distributions of ISWI and acetylated histone H4 are dissimilar on both autosomes and X chromosome, with few sites of overlap (arrows), as visible in the split-image magnifications. Distribution of ISWI [green] and acetylated histone H3 [red; K9Ac, K14Ac] on wild-type salivary gland polytene chromosomes [lower panel]. ISWI share a good number of sites on both autosomes and X chromosome with the acetylated histone H3 (arrows), as visible in the split-image magnifications. (B) Distributions and levels of acetylated histone H3 [upper pannel], acetylated histone H4 [middle panel] and Sin3A [lower panel] on DAPI stained polytene chromosomes from *w1118* strain[*wt*], *ISWI^1^/ISWI^2^* mutant [*ISWI*] and from salivary glands expressing wild-type ISWI [*uas-ISWI*]. The specificity of αH3Ac and αH4Ac antibodies has been tested by Western on unmodified and acetylated histone substrates (data not shown). Arrows indicate the puffed X chromosome on *ISWI^1^/ISWI^2^* mutant male chromosomes.

ISWI activity can be negatively regulated by the site-specific acetylation of its nucleosome substrate [29],[32]. Thus, the deacetylation of chromatin by the Sin3A/Rpd3 complex may promote ISWI-mediated remodeling. To test this model, we looked at the relative distributions of ISWI and acetylated histones H3 and H4 on wild-type salivary gland polytene chromosomes using antibodies that recognize specific acetylated residues of histone H3 and H4. ∼67% of ISWI bands are associated with chromatin that is hypoacetylated on histone H3 (Figure 7A), while, the great majority of ISWI (∼92%) is associated with chromatin that is hypoacetylated on histone H4 (Figure 7A). These data are in line with previous work showing that ISWI activity can be negatively regulated by site-specific histone acetylation [29],[32].

If the preferential association of ISWI with hypoacetylated chromatin results from its functional association with the Sin3A/Rpd3 histone deacetylase complex, one would predict that changes in the level of ISWI should affect the global levels and distribution of histone H3 and H4 acetylation on polytene chromosomes. We therefore examined whether the reduction in the levels of chromatin-bound Sin3A/Rpd3 histone deacetylase complex on the polytene chromosomes of *ISWI* mutants was accompanied by an increase in the levels or distributions of histone H3 and H4 acetylation (Figure 7B). While the overall level and distribution of acetylated histone H3 were comparable to wild-type chromosomes, a slight increase in the distribution of histone H4 acetylation was observed on *ISWI* mutant polytene chromosomes (Figure 7B). By contrast, polytene chromosomes from larvae over-expressing wild-type ISWI (Figure S7) show a reduction in the levels of histone H3 acetylation and a significant loss of acetylation associated with histone H4 (Figure 7B). Remarkably, the changes in histone acetylation resulting from ISWI misexpression are mirrored by changes in the levels of chromatin-bound Sin3A (Figure 7B).

The most pronounced changes in global histone acetylation result from the over-expression of ISWI, possibly because the overloading of wild type ISWI on polytene chromosomes could recruit the Sin3A/Rpd3 HDAC activity to a great number of chromatin loci. While the modest changes in histone acetylation observed in ISWI mutant chromosomes could result from residual ISWI activity of maternal source [13] or could be masked by the reactivity of the αH3Ac an αH4Ac antibodies for lysine residues whose acetylation is not sensitive to loss of *ISWI*. Our data indicate that changes in global histone acetylations resulting from altered levels of ISWI could be explained though a functional interaction between ISWI and the HDAC activity of Sin3A/Rpd3 at specific chromatin sites.

## Discussion

### ISWI Genetically Interacts with a Broad Range of Cellular and Nuclear Factors

Biochemical studies have provided a wealth of data concerning the mechanisms of action of ISWI but lack the complexity of chromatin that exists *in vivo*. To circumvent this problem, we conducted an unbiased genetic screen for regulators of ISWI function in Drosophila. Our screen produced the first genetic interaction map for the ATP-dependent chromatin remodeler ISWI in higher eukaryotes. We found that ISWI genetically interacts with a network of cellular and nuclear factors that escaped previous biochemical analyses, indicating the participation of ISWI in variety of biological processes (Figure S2 and Table S1A).

Interestingly, unbiased genetic screens aimed at the identification of factors involved in the regulation of vulval cell fates in *C.elegans* and sensory neuron morphogenesis in *Drosophila* have identified *ISWI* and some of our *ISWI^K159R^* enhancers as key regulators of these biological processes [45],[47]. Our GO analysis indicates ‘neuron differentiation’ and ‘cell cycle regulation’ as overrepresented categories within the combined strong and medium *ISWI^K159R^* enhancers. With hindsight this result is not surprising considering that our screen targeted the eye, an organ whose development is tightly linked to nervous system differentiation and the spatial as well as temporal control of cell division. Therefore, it is likely that some of the *ISWI^K159R^* enhancers we isolated could work in concert with ISWI to support the differentiation and development of the adult fly eye.

One of the goals of our screen was to isolate factors encoding enzymatic activities that could play a role in the regulation of ISWI *in vivo* by modifying ISWI or chromatin components with which ISWI interacts. As expected, our screen led to the isolation of a group of genes that includes kinases (e.g. *trbl, grp, snf4ag*), ATPases (e.g.; *pont*), proteins associated with deacetylases (*Sin3A*), methyl binding factor (*mbf1*) and enzymes regulating the metabolism of poly-ADP-ribose *(Parp)* (Table S1A). The variety of chromatin components we found in the screen indicates that it is likely that a functional cross talk exists between ISWI and other chromatin-remodeling and modifying activities working in the nucleus.

### ISWI Genetically and Physically Interacts with Sin3A and Rpd3

We found that *Drosophila ISWI* genetically interacts with *Sin3A* and with its associated histone deacetylase subunit Rpd3. This genetic interaction may reflect a physical interaction between ISWI, Sin3A and Rpd3, since the three proteins co-localizes at many, though not all, sites on polytene chomosome. Although the resolution of polytene chromosome staining is limited, our biochemical data are consistent with a physical interaction between ISWI and Sin3A/Rpd3 in embryo and larval stages. Previous biochemical studies in flies have not detected the presence of Sin3A and Rpd3 proteins as integral subunits of Drosophila ISWI complexes [5],[6]. Therefore, the physical interaction we found between ISWI and Sin3A/Rpd3 could be transient or indirect.

We found that the nucleosome stimulated ATPase activity of ISWI co-purifies with a histone deacetylase activity associated with the Sin3A/Rpd3 complex in larvae. Interestingly changes in the levels of ISWI alter the binding of Sin3A/Rpd3 to polytene chromosomes and are correlated with changes in global histone H3 and H4 acetylation. Because ISWI function can be antagonized by the site-specific acetylation of histones [29]–[32], it is possible that the Sin3A/Rpd3 complex positively regulates ISWI activity *in vivo*. Therefore, ISWI and the Sin3A/Rpd3 complex may facilitate each other's function, forming a positive feedback system for chromatin regulation.

Genetic and biochemical studies in yeast have shown that the nucleosome spacing activity of the Isw2 complex can repress transcription in a parallel pathway with the yeast Sin3/Rpd3 histone deacetylase complex [48],[49]. Although, the functional organization of DNA into chromatin is conserved among eukaryotes, mutations in the two yeast counterparts of ISWI, *Isw1* and *Isw2*, do not show any severe phenotype [48],[49]. Conversely, ISWI is a unique and essential gene in *Drosophila* highlighting a possible divergent role for ISWI in flies and a distinct mechanism of interaction with the Sin3A/Rpd3 complex in higher eukaryotes. Indeed, interactions between SNF2L, a mouse ISWI homolog, and the Sin3A/Rpd3 complex have been proposed to play a role in repressing ribosomal gene transcription in mammals [17],[18]. Furthermore, studies of the thymocyte-enriched chromatin factor SAT1B indicate that its ability to regulates gene expression and organize chromatin folding into loop domains at the IL-2Ra locus is dependent on the catalytic activities of Sin3A/HDAC1 (the mammalian Rpd3) and the ISWI homolog SNF2H protein [54].

ISWI can also be a target of site-specific acetylation by the GCN5 histone acetyltransferase [34]. Therefore, the functional association we found between ISWI and Sin3A/Rpd3 could help regulate the acetylation state of ISWI and modulate its activity. Interestingly, it has been recently reported that *ISWI* genetically interact with the histone acetyltransferase GCN5 [55]. *gcn5* mutations cause chromosome condensation defects very similar to the one observed in *ISWI* and *E(bx)* mutants, as well as global loss of histone H4 acetylation on lysine 12 [55]. A decrease in ISWI activity as a consequence of loss of GCN5-dependent acetylation could in theory account for the observed defects. An alternative possibility is that specific histone acetylations differently regulate ISWI function. Therefore, further studies will be necessary to clarify the roles of Sin3A, Rpd3 and other histone modifying enzymes in the regulation of ISWI-containing complexes function *in vivo*.

## Materials and Methods

### *Drosophila* Stocks, Genetic Crosses and DNA Constructs

Flies were raised on cornmeal/sucrose/yeast/agar medium containing Tegosept. Unless otherwise indicated, *Drosophila* strains were obtained from Bloomington Stock Center and are described in FlyBase (www.flybase.org). The collection of EP lines [40],[41] was obtained from Exelixis (http://Drosophila.med.harvard.edu/). To monitor the effect of the *ISWI^K159R^* transgene misexpression on chromosome structure of eye disc cells, *P[w^+^, ey-GAL4], P[w^+^, UASGALhsp70:ISWI^K159R^]/ P[w^+^, UAS-GFP.nls]8* larvae were generated by crossing homozygous *w/Y; P[w^+^, UAS-GFP.nls]8* males with *y w, P[w^+^, ey-GAL4], P[w^+^, UASGALhsp70:ISWI^K159R^]/ T(2:3) CyO;TM6B Tb* virgins [38] and recognized by the absence of the dominant Tb marker. Trans-heterozygous *ISWI^1^/ISWI^2^* male larvae [7], for immuno-fluorescence analysis, were obtained by crossing *w/Y; ISWI^1^, Bc/ SM5, Cy* males to *y w; ISWI^2^/T(2:3) CyO;TM6B Tb* virgin females and were recognized by their yellow mouth hooks, the presence of the dominant Bc marker and the absence of the Tb marker. To misexpress wild-type ISWI in the salivary glands, we crossed *w; P[w^+^, UASGALhsp70:ISWI]* males [7] with y *w, P[w^+^, ey-GAL4]* virgins [56]. The pUAST-ISWI-CTAP DNA construct was obtained by cloning the Pfu-amplified ISWI cDNA into the Not I and Xho I sites of the pUAST-CTAP vector polylinker [57]. The pUAST-ISWI-CTAP DNA construct was injected in y w embryos to generate germline transformants as described elsewhere [58]. Larvae expressing TAP-tagged ISWI were obtained crossing y w, P[w^+^, Act5C-GAL4] virgins with y w, P[w^+^, UASGALhsp70:ISWI-CTAP] males.

### Analysis of Polytene and Mitotic Chromosomes

Wild-type Oregon R and *ISWI^1^/ISWI^2^* mutant polytene chromosomes were prepared from third-instar larvae grown at 18°C and identified using the larval markers y, Bc and Tb. Primary antibodies used for immuno-stainings include rabbit antibodies against ISWI [59], Sin3A [35], Rpd3 [35], Mod [52] and acetylated histone H3 and H4 (Upstate; specifically recognizing histone H3 acetylations on K9 and K14 and histone H4 acetylations on K5, K8, K12 and K16). For single staining polytene chromosomes were processed as described previously [7],[35]. The Fab fragment blocking method was used to stain polytene chromosomes with two primary antibodies raised in rabbits [60]. Immuno-staining with the monoclonal antibody LA9 directed against the Mod protein was performed as previously described [52]. Staining with H3Ac and H4Ac antibodies was done using the citric acid/methanol/acetone fixation described previously [7]. Mitotic metaphase chromosomes from eye disc cells were obtained as previously described [61]. Images were captured with a DC300F camera on a Leica DM IRB microscope. Quantitative densitometric analysis was performed using the QFluor (Leica) software. Split images were generated as described previously [60].

### Genetic Screen for dominant modifiers of *ISWI^K159R^*

To screen for dominant modifiers of *ISWI^K159R^* eye phenotypes, single crosses between three males coming from EP line insertions on the second or third chromosomes and three virgins of the sensitized w, P[w^+^, ey-GAL4], P[w^+^, UASGALhsp70:ISWI^K159R^]/TM3, Sb recombinant line [7] were set at 25°C. For EP insertions on the X chromosome, three virgins of each EP line were crossed at 25°C with three w, P[w^+^, ey-GAL4], P[w^+^, UASGALhsp70:ISWI^K159R^]/TM3, Sb males. The resulting F_1_ progeny was screened for eye defects using a 1 to 6 scale, which allows a statistical representation of the range of eye phenotype resulting from the misexpression of the *ISWI^K159R^* transgene [37],[38]. An EP line mutation was selected as an enhancer of *ISWI^K159R^* if the eye score distribution of the experimental F_1_ progeny was centered on class 4, while the control class was centered on class 2. In particular, we defined strong, medium and weak enhancers as follows: (strong enhancers) if the frequency of class 4 was >50%; (medium enhancers) if the frequency of class 4 was between 30–50%; (weak enhancers) if the frequency of class 4 was between 15–30%. False *ISWI^K159R^* enhancers were identified by the presence of eye defects in the F_1_ progeny coming from crosses between EP line males and homozygous w, P[w^+^, ey-GAL4] virgins. As a specificity control, enhancer of *ISWI^K159R^* EP line mutations were assayed for their ability to modify eye defects caused by expression of *brm^K804R^* in the developing eye.

### Bioinformatic Analysis

For the Biogrid analysis [46], each *ISWI^K159R^* enhancer EP line was associated to a single gene based on the insertion DNA sequence data available on flybase (www.flybase.org). The gene ontology data and all the genetic and physical interactions existing between the *ISWI^K159R^* enhancer EP line were obtained from the Biogrid website (www.thebiogrid.org) and represented in a graphical format using the Osprey software [62] (http://biodata.mshri.on.ca/osprey/servlet/Index). For the GO analysis the gene annotation (Oct 27, 2007) and the Gene Ontology (Oct 1, 2007) provided by FlyBase were used with Ontologizer [43] to determine overrepresented GO terms in the strong, medium and weak enhancers sets as compared to the entire EP library. The parent-child method of Ontologizer, which takes into account the parent-child relationships of the GO hierarchy, was applied and the P-values were adjusted using Westfall-Young Single-Step multiple testing correction. A corrected P-value threshold of 0.1 was used as a cut-off for reporting significant matches. To compute statistical significance of the frequencies of GO-component terms hypergeometric distributions were calculated based on the occurrence of the indicated terms and corrected for multiple testing by the Bonferroni correction method.

### Protein Extracts

Native protein extracts from 0–16 hours embryos were prepared as described [63]. Salivary glands protein extracts from the HA-6His-tagged ISWI line [7], *w1118*, *ISWI^1^/ISWI^2^* mutants, and P[w^+^, ey-GAL4]; P[w^+^, UASGALhsp70:ISWI] third instar larvae were obtained dissecting 20 pair of glands in 0.7% NaCl following vortexing in 2 µl/glands of RIPA buffer (1% NP40, 0.5% sodium deoxicolate, 0.1% SDS in PBS). The homogenate was centrifuged at 20000 g for 30 min and the supernatant was flash frozen in liquid nitrogen and stored at −80°C. Native larval nuclear protein extracts were prepared as described elsewhere [50].

### Immunoprecipitations

Co-immunoprecipitation on embryo protein extracts derived from flies carrying an HA-6His-tagged ISWI [7] was performed as described previously [63] using the rat *3F10* monoclonal anti-HA antibody (Roche). Co-immunoprecipitation using anti-ISWI was performed using the ‘Catch and Release Kit’ (Upstate 17-500) with 500 µg of wild-type embryo protein extracts incubated with 4 µg of anti-ISWI [64] or generic IgG (Santa Cruz Biotechnology) as negative control. Western blotting analysis was conducted with the ChemiDoc XRS imager (BioRad).

### Chromatography

#### His Trap and Gel Filtration

40 ml of larval nuclear extracts derived from the HA-6His-tagged ISWI line [7] were injected in a 1ml HisTrap HP affinity column (G&E Healthcare) and subjected to standard nickel-coupled affinity resin chromatography. About 400 µl of ISWI-enriched fractions from the HisTrap column were loaded on a Superpose-6 column pre-equilibrated with HisTrap Binding buffer without imidazole (15 mM Hepes-KOH pH 8, 250 mM KCl, 10% glycerol).

#### Affinity capture of TAP-tagged ISWI

60 ml of nuclear extracts derived from TAP-tagged ISWI expressing larvae was exchanged with IPP150 buffer (10 mM Tris–Cl, pH 8.0, 150 mM NaCl, 0.1% Nonidet) on HiPrep 26/10 desalting columns (G&E Healthcare). TAP-epitope tagged ISWI was pulled-down using published standard protocols [65].

### Enzymatic Assays

Aliquots of 2 µl from the Superose-6 column fractions or 4 µl from the TAP-tagged purified material was assayed for nucleosome-stimulated ATPase activity, as previously published [7],[39], in the presence of 5 µCi [γ-^33^P]ATP - 3000 Ci mmol-1 (G&E Health Care) and 100 ng of *in vitro* assembled chromatin [66] were separated by thin layer chromatography on TLC cellulose plates (Merk). Quantification of ATP hydrolysis was done with the Personal Molecular Imager FX System (BioRad).

To monitor HDAC activity, recombinant *Drosophila* core histones [67] were labeled with [^3^H]-Acetyl-CoA (G&E Healthcare) using recombinant MOF [68] and PCAF (Upstate) as HAT sources. Acetyl transfer to histones was detected by fluorography as described [68]. To monitor HDAC activity, aliquots of 100 µl of the Superose-6 column fractions or 25 µl of the TAP-tagged purified material were assayed as previously reported [69] . The released [^3^H] acetate was counted on a Beckman LS1801 detector by liquid scintillation.

## Supporting Information

Figure S1 **Missexpression of** ***ISWI^K159R^*** **in the developing eye discs causes chromosome condensation defects.** (A and B) The expression territories of the *ey* gene and (E and F) the *UAS-ISWI^K159R^* transgene in the developing eye disc were indirectly monitored by misexpression of the *UAS-GFP* transgene with the *ey-GAL4* driver. According to the *ey* expression pattern in the eye discs [70], the *ISWI^K159R^* transgene is expressed posteriorly in the cycling cells before the morphogenetic furrow and it appears at the time of photoreceptor determination. (A and B) While control eye discs show a normal pattern of developing photoreceptors, (E and F) the eye territories expressing *ISWI^K159R^* show defects in the organization of photoreceptor clusters. (C) While the expression of the *UAS-GFP* transgene has no effect on chromosome structure, (G and H) the expression of *ISWI^K159R^* in eye disc cells causes dramatic mitotic chromosome defects in eye disc cells. In particular, the ISWI^K159R^ expressing nuclei in the eye-antennal discs produce chromatin that resolves into aberrant methaphases with severe chromosomes condensation defects. The misexpression of the *ISWI ^K159R^* transgene in the eye disc cells causes chromosome condensation defects that probably contribute to the observed adult eye phenotypes. (I) Expression of the *UAS-ISWI^K159R^* transgene, (D) but not *UAS-GFP*, in the developing eye using an *ey*-GAL4 driver has strong effects on cell viability and results in flies with rough and reduced eyes [7].
(2.06 MB TIF)

Figure S2 **ISWI genetically interacts with a wide range of cellular components.** (A) The 255 genes corresponding to *ISWI^K159R^* enhancers EP line loci are depicted as nodes, colored according to their current gene ontology (GO) categories, as indicated to the right. Numbers between brackets indicate the frequency of that GO term in the EP library followed by its frequency amongst the *ISWI^K159R^* enhancers. (B) Same diagram as in (A) except that nodes are colored according to the strength with which the corresponding EP lines enhanced *ISWI^K159R^* eye phenotypes. Although we have used highly selective secondary screens to identify ISWI specific interactors, we cannot exclude that some of the weak eye phenotype enhancements we recovered could be the result of general cell stress independently imposed to the developing eye disc by the simultaneous presence of the EP and the overexpressing *ISWI^K159R^* transgenes. (C) Intracellular localization of the gene products encoded by the 255 *ISWI^K159R^* enhancer loci. The Ras85D node is indicated because it concentrates 55% of all the genetic interactions amongst the 255 *ISWI^K159R^* enhancers. The edges represent known physical and genetic interactions.
(0.89 MB TIF)

Figure S3 **Gene Ontology analysis of** ***ISWI^K159R^*** **interacting EPs.** (A) GO terms representation of the entire EP line collection as compared to the fly proteome. To determine enrichment of the EP library, genes hit in the EP collection were compared to all Drosophila genes. (B) Overrepresented GO terms in the strong, and combined strong/medium *ISWI^K159R^* enhancers as compared to the entire EP collection. Specific GO terms can be visualized by image zooming. A corrected P-value threshold of 0.1 was used as a cut-off for reporting significant matches.
(0.05 MB PDF)

Figure S4 **HisTrap coupled to Size fractionation of larval nuclear extract.** (A) ISWI-enriched fractions from the HisTrap column, corresponding to ∼1/400 of the unbound extract, were size fractionated on a Superpose-6 gel filtration column. ISWI together with Sin3A and Rpd3 elute in fractions of high molecular weight of about 600 KDa. Western blot analysis was performed on 5% of the total input extract [I] and collected fractions, using antibodies against ISWI, Sin3A and Rpd3. (B) The Superose-6 fractions were assayed for nucleosome-stimulated ATPase and (C and D) HDAC activity on acetylated histone H4 and H3 substrates. The fractions enriched in ISWI showed specific nucleosome stimulated ATPase and histone H4 and H3 HDAC activity. For the ATPase assay, 0.5% of Input [I] and Superose-6 fractions were tested for ATPase activity in the presence of 100 ng of reconstituted recombinant chromatin. The HDAC assays were conducted on 15000 cpm of acetylated histones with a mock input [M], with 20% of Input [I] and Superose-6 fractions in the presence and absence of the HDAC inhibitor sodium butirrate [NaB].
(2.37 MB TIF)

Figure S5 **ISWI interaction with Sin3A/Rpd3 in salivary glands and characterization of acetylated histone substrates and gel filtration fractions used for ATPase and HDAC assays.** (A) Immunoprecipitation with anti-HA antibodies on salivary gland total protein extracts derived from a line expressing HA-tagged ISWI (HA-ISWI) and from control extracts (ISWI). ISWI is specifically immunoprecipitated from the HA-ISWI extract together with the Rpd3 and Sin3A proteins. Western blot analysis was performed on 10% of the total input extract [I], supernatant [S], wash [W], and 30% of the total pellet [P] using antibodies against ISWI, Sin3A and Rpd3. (B) SDS PAGE showing the integrity and purity of the full-length MOF stained by Coomassie. Recombinant Drosophila histone octamers acetylated with [^3^H]-Acetyl-CoA (C) by MOF or (D) by PCAF were separated by SDS PAGE [lane 1] and visualized by fluorography [lane 2]. (E) Immunoprecipitation with anti-HA antibodies on gel filtration fractions with high [#25] and low [#33] nucleosome-stimulated ATPase and HDAC activities. ISWI is specifically pulled down from fraction #25 [lane 3] together with Sin3A and Rpd3. Input [I].
(2.84 MB TIF)

Figure S6 **Quantification and staining of Sin3A and Rpd3 on** ***ISWI*** **mutant Chromosomes.** (A) Salivary glands protein extracts from *ISWI^1^/ISWI^2^* mutants [*ISWI*] and the *w1118* strain [*wt*] were assayed by Western blotting with antibodies against, Sin3A, ISWI, Rpd3, and αTubulin. In the ISWI mutant extracts the level of the Rpd3 protein does not change relative to αTubulin. Although, we find a reduction in the level of chromatin bound Sin3A in ISWI mutant chromosomes, the Sin3A protein appear to be more abundant in *ISWI* mutants then in *wt* total salivary gland protein extracts. (B) To control for uniform antibody accessibility to chromosomes and to exclude a general loss of chromatin bound proteins we compared the binding of the chromatin Mod protein in wild-type and *ISWI* mutant chromosomes. The anti-Mod antibody stains with comparable intensity the nucleolus (arrowheads) and many bands on polytene chromosomes on both wild-type (wt) and *ISWI* mutant chromosomes. The DAPI stained *ISWI* mutant male X chromosome is indicated by an arrow. (C) Quantification of Sin3A and (D) Rpd3 staining levels in double immunostainings for Sin3A/Mod and Rpd3/Mod in wild type [wt] and ISWI mutant [*ISWI*] chromosomes, using the Mod signal as internal control [52],[53].
(1.08 MB TIF)

Figure S7 **Over-expression of ISWI on polytene chromosomes.** (A) DAPI staining and distribution of ISWI on polytene chromosome from salivary glands misexpressing wild-type ISWI (UAS-ISWI) using an *eyGAL4* driver, and on control *w1118* chromosomes (wt). Salivary gland cells misexpressing ISWI have polytene chromosomes overloaded with ISWI. Images were captured using identical exposure settings. (B) Western blot analysis confirms that there is about 50 fold more ISWI on protein extracts from salivary glands expressing ISWI [lane 2] than in control salivary glands from the *w1118* strain [lane 1].
(1.08 MB TIF)

Table S1 (A) *ISWI^K159R^* Screening data. *ISWI^K159R^* enhancer EPs mapped to specific gene loci based on the iPCR data available on Flybase. The strength of interaction has been calculated as described in methods. Interaction with *brm^K804R^* and microarray expression data [13] in male and female salivary glands (SG) or whole larvae (WL) are shown in separate columns. (B) *False positives.* False *ISWI^K159R^* enhancers are listed and sorted by the strength of interaction resulting with the *eyGAL4* driver. (C) *Multiple hits.* Genes that were hit by multiple EP insertions are listed together with their interaction strengths.
(0.07 MB XLS)
