# Supplementary figures and images for: Genetic Identification of a Network of Factors that Functionally Interact with the Nucleosome Remodeling ATPase ISWI
Source: PLoS Genet. 2008 Jun 6;4(6):e1000089. doi: 10.1371/journal.pgen.1000089 (PMC2390755; doi:10.1371/journal.pgen.1000089)

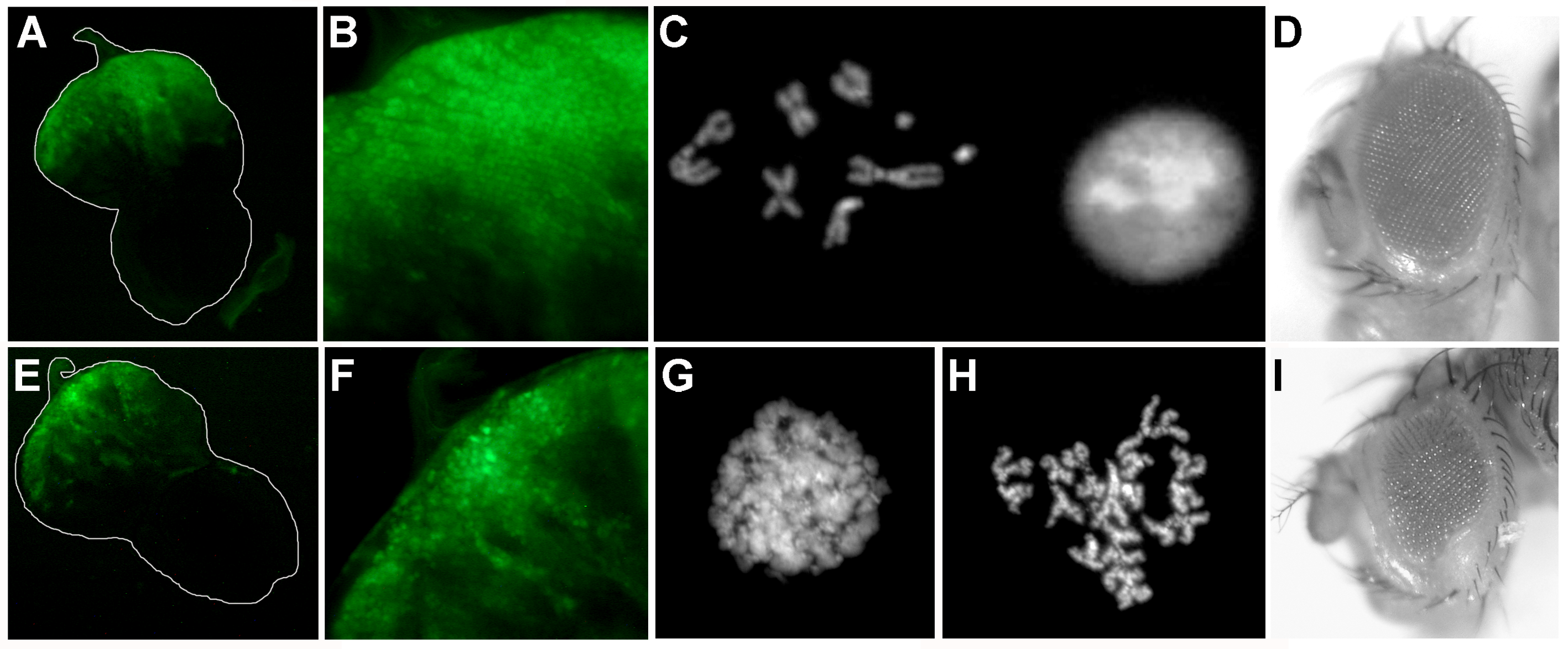

Supplement: Figure S1 — Missexpression of ISWIK159R in the developing eye discs causes chromosome condensation defects. (A and B) The expression territories of the ey gene and (E and F) the UAS-ISWIK159R transgene in the developing eye disc were indirectly monitored by misexpression of the UAS-GFP transgene with the ey-GAL4 driver. According to the ey expression pattern in the eye discs [70], the ISWIK159R transgene is expressed posteriorly in the cycling cells before the morphogenetic furrow and it appears at the time of photoreceptor determination. (A and B) While control eye discs show a normal pattern of developing photoreceptors, (E and F) the eye territories expressing ISWIK159R show defects in the organization of photoreceptor clusters. (C) While the expression of the UAS-GFP transgene has no effect on chromosome structure, (G and H) the expression of ISWIK159R in eye disc cells causes dramatic mitotic chromosome defects in eye disc cells. In particular, the ISWIK159R expressing nuclei in the eye-antennal discs produce chromatin that resolves into aberrant methaphases with severe chromosomes condensation defects. The misexpression of the ISWI K159R transgene in the eye disc cells causes chromosome condensation defects that probably contribute to the observed adult eye phenotypes. (I) Expression of the UAS-ISWIK159R transgene, (D) but not UAS-GFP, in the developing eye using an ey-GAL4 driver has strong effects on cell viability and results in flies with rough and reduced eyes [7]. (2.06 MB TIF) [file pgen.1000089.s001.tif]

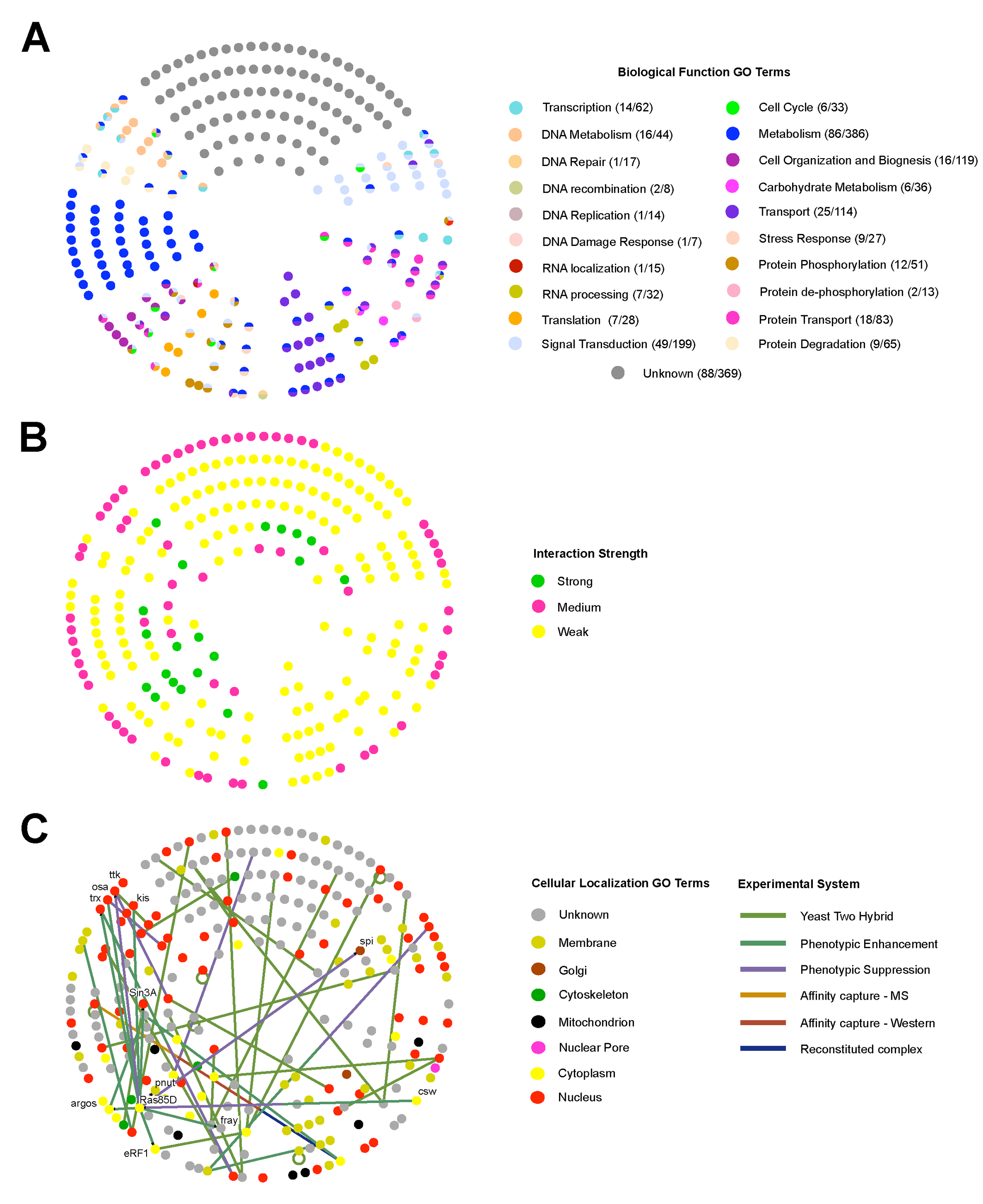

Supplement: Figure S2 — ISWI genetically interacts with a wide range of cellular components. (A) The 255 genes corresponding to ISWIK159R enhancers EP line loci are depicted as nodes, colored according to their current gene ontology (GO) categories, as indicated to the right. Numbers between brackets indicate the frequency of that GO term in the EP library followed by its frequency amongst the ISWIK159R enhancers. (B) Same diagram as in (A) except that nodes are colored according to the strength with which the corresponding EP lines enhanced ISWIK159R eye phenotypes. Although we have used highly selective secondary screens to identify ISWI specific interactors, we cannot exclude that some of the weak eye phenotype enhancements we recovered could be the result of general cell stress independently imposed to the developing eye disc by the simultaneous presence of the EP and the overexpressing ISWIK159R transgenes. (C) Intracellular localization of the gene products encoded by the 255 ISWIK159R enhancer loci. The Ras85D node is indicated because it concentrates 55% of all the genetic interactions amongst the 255 ISWIK159R enhancers. The edges represent known physical and genetic interactions. (0.89 MB TIF) [file pgen.1000089.s002.tif]

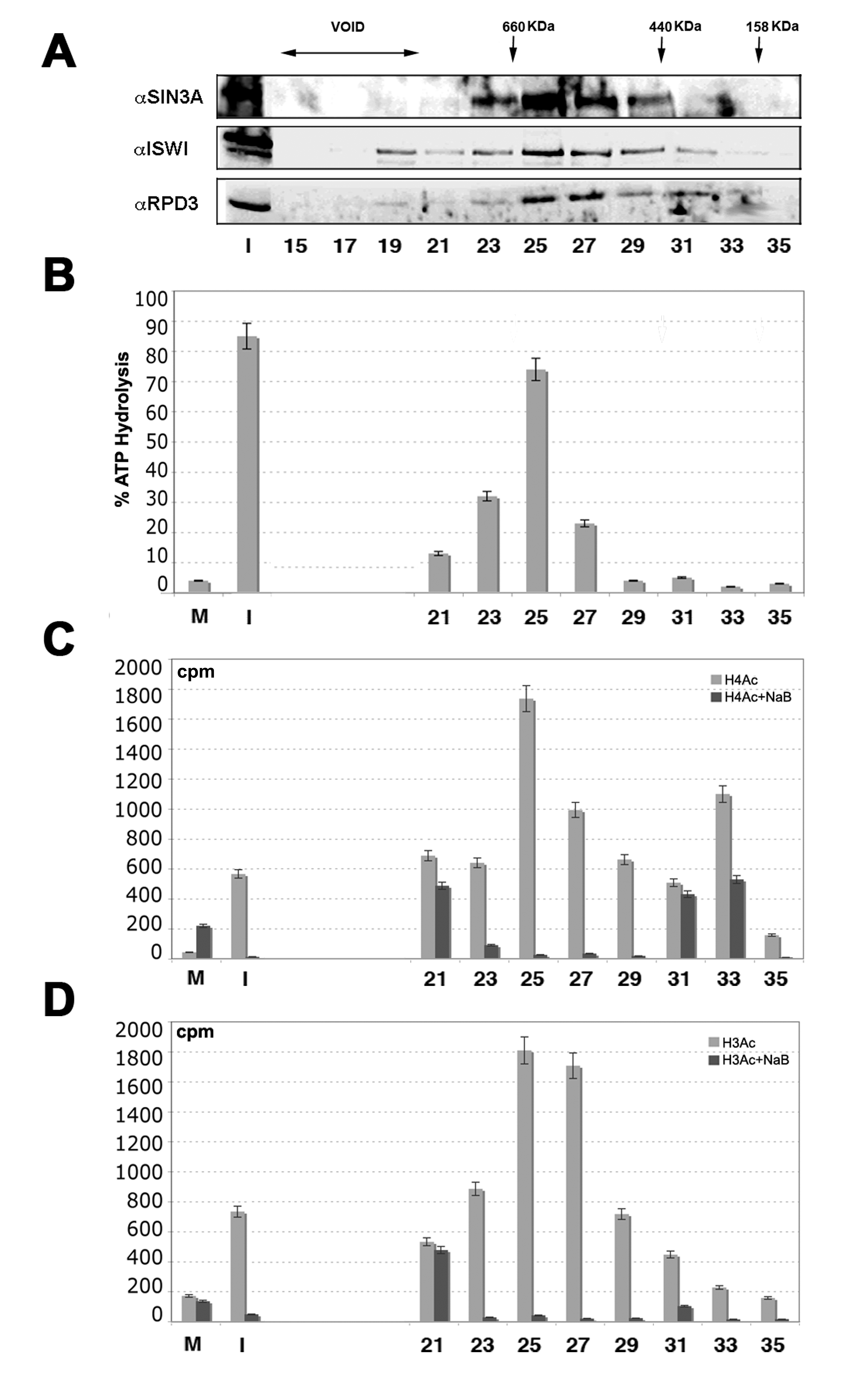

Supplement: Figure S4 — HisTrap coupled to Size fractionation of larval nuclear extract. (A) ISWI-enriched fractions from the HisTrap column, corresponding to ∼1/400 of the unbound extract, were size fractionated on a Superpose-6 gel filtration column. ISWI together with Sin3A and Rpd3 elute in fractions of high molecular weight of about 600 KDa. Western blot analysis was performed on 5% of the total input extract [I] and collected fractions, using antibodies against ISWI, Sin3A and Rpd3. (B) The Superose-6 fractions were assayed for nucleosome-stimulated ATPase and (C and D) HDAC activity on acetylated histone H4 and H3 substrates. The fractions enriched in ISWI showed specific nucleosome stimulated ATPase and histone H4 and H3 HDAC activity. For the ATPase assay, 0.5% of Input [I] and Superose-6 fractions were tested for ATPase activity in the presence of 100 ng of reconstituted recombinant chromatin. The HDAC assays were conducted on 15000 cpm of acetylated histones with a mock input [M], with 20% of Input [I] and Superose-6 fractions in the presence and absence of the HDAC inhibitor sodium butirrate [NaB]. (2.37 MB TIF) [file pgen.1000089.s004.tif]

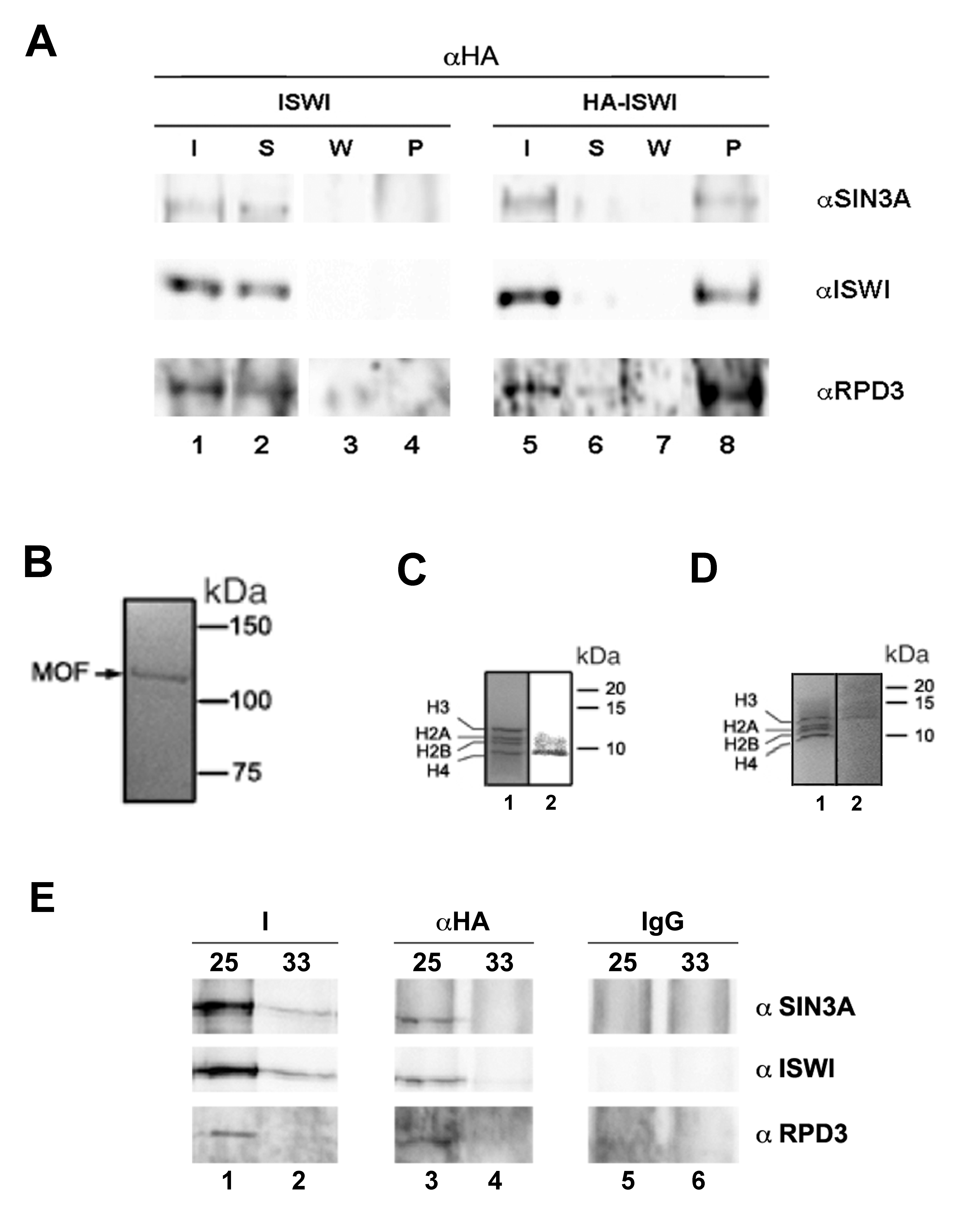

Supplement: Figure S5 — ISWI interaction with Sin3A/Rpd3 in salivary glands and characterization of acetylated histone substrates and gel filtration fractions used for ATPase and HDAC assays. (A) Immunoprecipitation with anti-HA antibodies on salivary gland total protein extracts derived from a line expressing HA-tagged ISWI (HA-ISWI) and from control extracts (ISWI). ISWI is specifically immunoprecipitated from the HA-ISWI extract together with the Rpd3 and Sin3A proteins. Western blot analysis was performed on 10% of the total input extract [I], supernatant [S], wash [W], and 30% of the total pellet [P] using antibodies against ISWI, Sin3A and Rpd3. (B) SDS PAGE showing the integrity and purity of the full-length MOF stained by Coomassie. Recombinant Drosophila histone octamers acetylated with [3H]-Acetyl-CoA (C) by MOF or (D) by PCAF were separated by SDS PAGE [lane 1] and visualized by fluorography [lane 2]. (E) Immunoprecipitation with anti-HA antibodies on gel filtration fractions with high [#25] and low [#33] nucleosome-stimulated ATPase and HDAC activities. ISWI is specifically pulled down from fraction #25 [lane 3] together with Sin3A and Rpd3. Input [I]. (2.84 MB TIF) [file pgen.1000089.s005.tif]

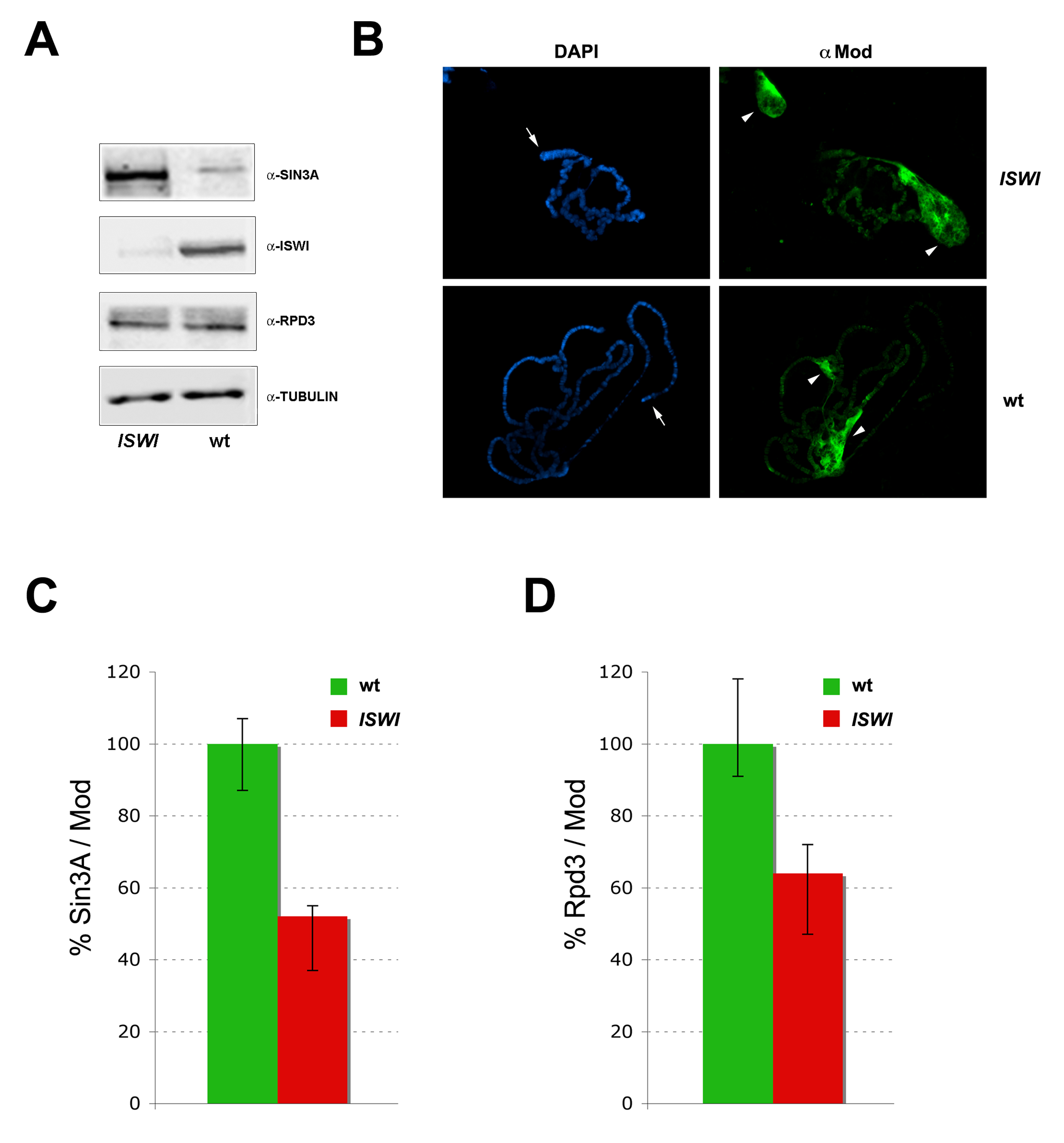

Supplement: Figure S6 — Quantification and staining of Sin3A and Rpd3 on ISWI mutant Chromosomes. (A) Salivary glands protein extracts from ISWI1/ISWI2 mutants [ISWI] and the w1118 strain [wt] were assayed by Western blotting with antibodies against, Sin3A, ISWI, Rpd3, and αTubulin. In the ISWI mutant extracts the level of the Rpd3 protein does not change relative to αTubulin. Although, we find a reduction in the level of chromatin bound Sin3A in ISWI mutant chromosomes, the Sin3A protein appear to be more abundant in ISWI mutants then in wt total salivary gland protein extracts. (B) To control for uniform antibody accessibility to chromosomes and to exclude a general loss of chromatin bound proteins we compared the binding of the chromatin Mod protein in wild-type and ISWI mutant chromosomes. The anti-Mod antibody stains with comparable intensity the nucleolus (arrowheads) and many bands on polytene chromosomes on both wild-type (wt) and ISWI mutant chromosomes. The DAPI stained ISWI mutant male X chromosome is indicated by an arrow. (C) Quantification of Sin3A and (D) Rpd3 staining levels in double immunostainings for Sin3A/Mod and Rpd3/Mod in wild type [wt] and ISWI mutant [ISWI] chromosomes, using the Mod signal as internal control [52],[53]. (1.08 MB TIF) [file pgen.1000089.s006.tif]

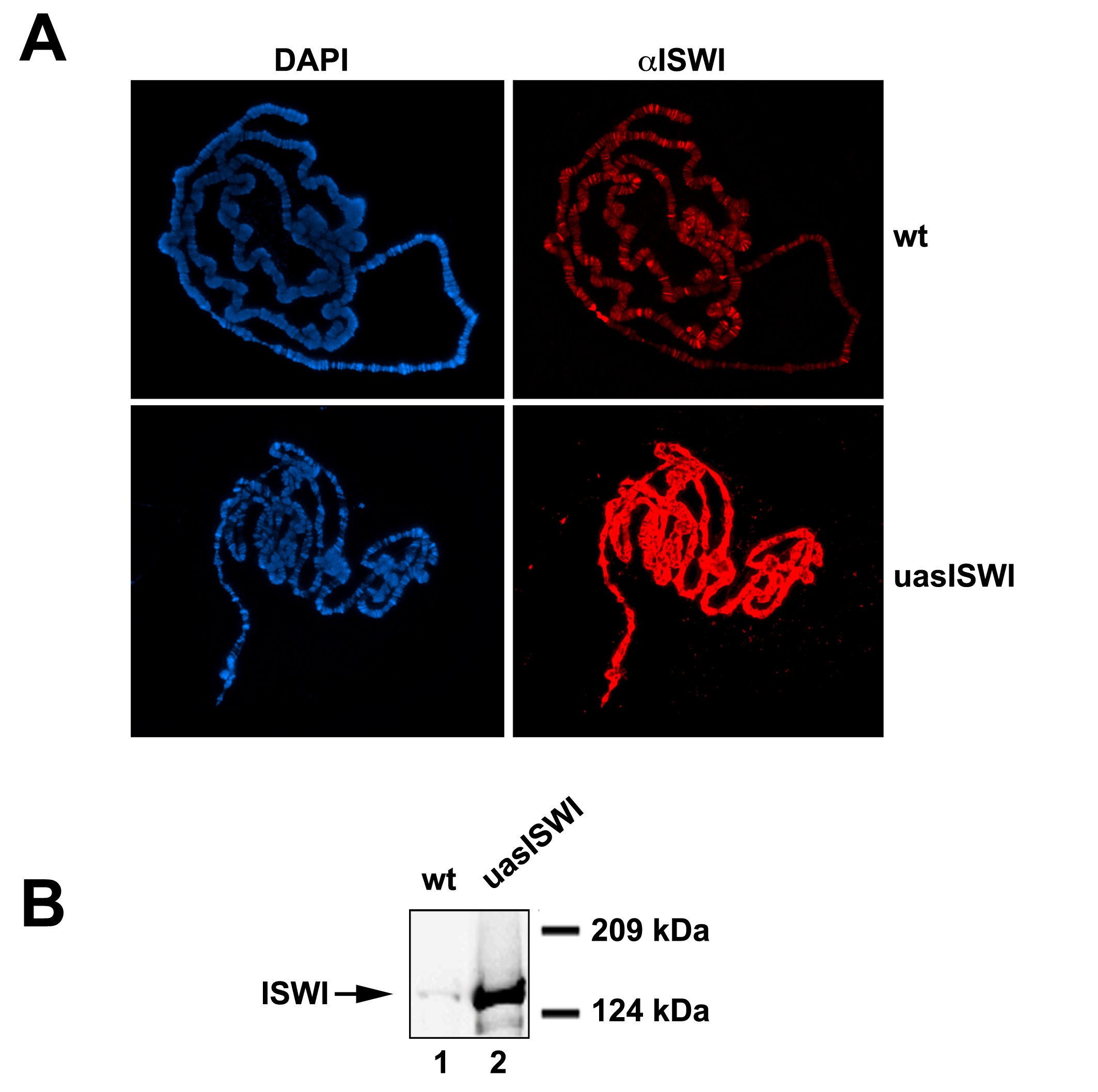

Supplement: Figure S7 — Over-expression of ISWI on polytene chromosomes. (A) DAPI staining and distribution of ISWI on polytene chromosome from salivary glands misexpressing wild-type ISWI (UAS-ISWI) using an eyGAL4 driver, and on control w1118 chromosomes (wt). Salivary gland cells misexpressing ISWI have polytene chromosomes overloaded with ISWI. Images were captured using identical exposure settings. (B) Western blot analysis confirms that there is about 50 fold more ISWI on protein extracts from salivary glands expressing ISWI [lane 2] than in control salivary glands from the w1118 strain [lane 1]. (1.08 MB TIF) [file pgen.1000089.s007.tif]
