# Supplementary material for: Genetic Identification of a Network of Factors that Functionally Interact with the Nucleosome Remodeling ATPase ISWI
Source: PLoS Genet. 2008 Jun 6;4(6):e1000089. doi: 10.1371/journal.pgen.1000089 (PMC2390755; doi:10.1371/journal.pgen.1000089)

A EP lines versus *Drosophila* proteome

B Strong *ISWI-K159R* enhancers versus EP lines

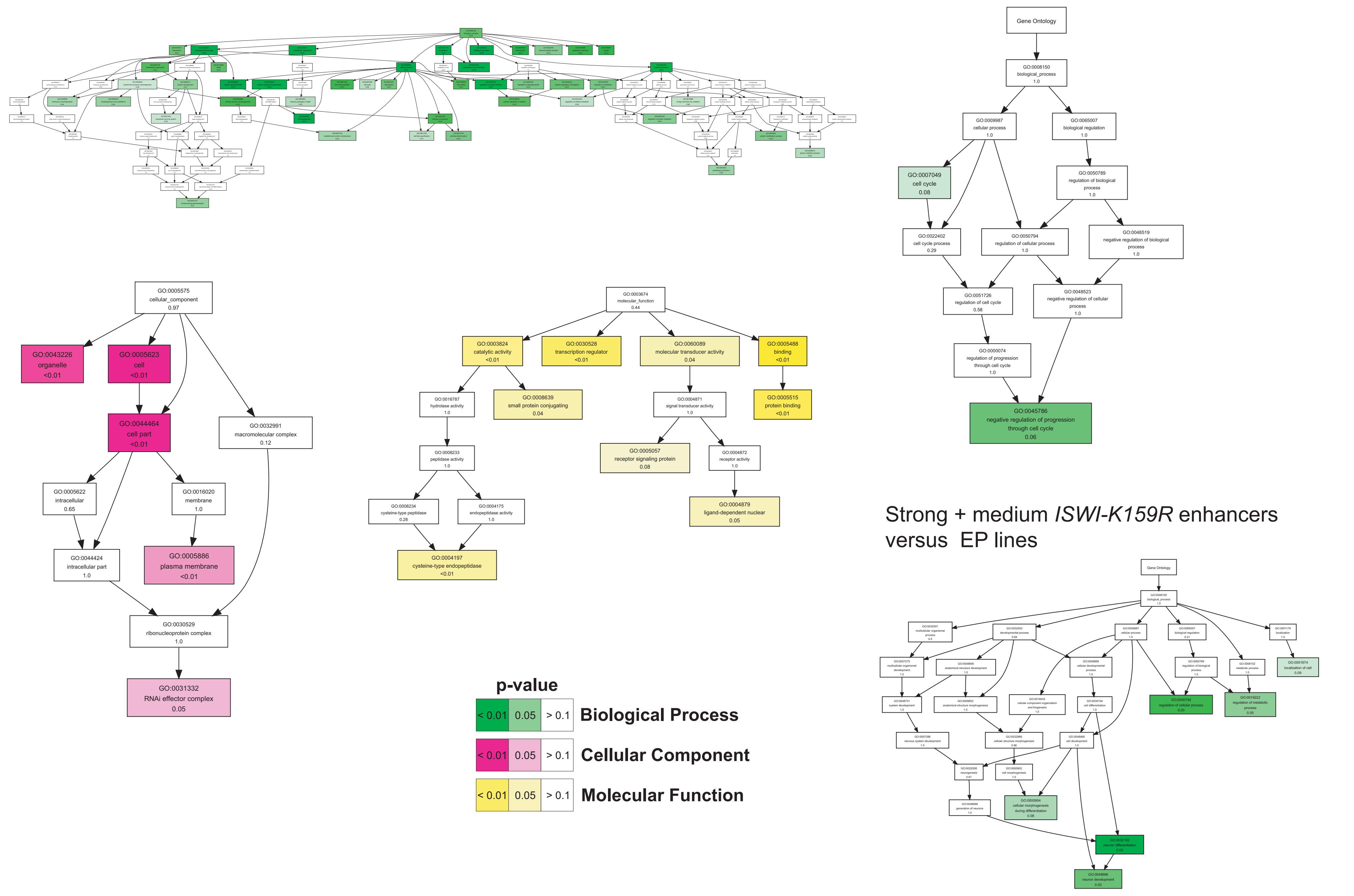

Supplement: Figure S3 — Gene Ontology analysis of ISWIK159R interacting EPs. (A) GO terms representation of the entire EP line collection as compared to the fly proteome. To determine enrichment of the EP library, genes hit in the EP collection were compared to all Drosophila genes. (B) Overrepresented GO terms in the strong, and combined strong/medium ISWIK159R enhancers as compared to the entire EP collection. Specific GO terms can be visualized by image zooming. A corrected P-value threshold of 0.1 was used as a cut-off for reporting significant matches. (0.05 MB PDF) [file pgen.1000089.s003.pdf]
